# Anatomical-Contextual YOLOv8–YOLOv12 Framework for Pulmonary Nodule Detection in CT: Multi-Organ Learning and Cross-Dataset Validation

**DOI:** 10.3390/diagnostics16182976

**Published:** 2026-09-14

**Authors:** Daniel Resendiz-Ventura, Moisés Márquez-Olivera, Viridiana Hernández-Herrera, Laura Marrujo-García, Antonio-Gustavo Juárez-Gracia, Irlanda Pacheco-Bravo

**Affiliations:** 1 Cyber-Physical Systems Laboratory, Centro de Investigación e Innovación Tecnológica (CIITEC), Instituto Politécnico Nacional (IPN), Cerrada Cecati s/n Col. Sta. Catarina, Azcapotzalco, Ciudad de México C.P. 02250, Mexico; lresendizv2400@alumno.ipn.mx; 2 Centro de Investigación en Ciencia Aplicada y Tecnología Avanzada, Unidad Legaria (CICATA Legaria), Instituto Politécnico Nacional (IPN), Av. Legaria No. 694 Col. Irrigación, Miguel Hidalgo, Ciudad de México C.P. 11500, Mexico; 3 CAPROM Applied Science to Mexican Problems, Venustiano Carranza 90, Azcapotzalco, Ciudad de México C.P. 02440, Mexico; lmarrujog@caprom.mx; 4 UMAE Hospital de Oncología, Centro Médico Nacional Siglo XXI, IMSS, A. Cuauhtemoc 330, Col. Doctores, Alcaldia Cuauhtemoc, Ciudad de México C.P. 06720, Mexico; drairlandapacheco@hotmail.com

**Keywords:** computed tomography (CT), pulmonary nodule detection, anatomical-contextual learning, multi-organ learning, ablation study, cross-dataset generalization, YOLO, deep learning, computer-aided diagnosis, LUNG-PET-CT-DX, LIDC-IDRI

## Abstract

**Background/Objectives:** Lung cancer outcomes depend strongly on early detection, highlighting the need for accurate, robust, and interpretable methods for identifying pulmonary nodules on computed tomography (CT). This study proposes a multi-organ anatomical-contextual framework based on YOLOv8–YOLOv12, in which supporting thoracic structures are incorporated as spatial references during training while the pulmonary nodule is retained as a single invariant pathological class. **Methods:** The models were trained and internally validated on 5000 2D axial CT slices from 320 patients in the LUNG-PET-CT-DX dataset and externally evaluated on an independent LIDC-IDRI subset comprising 700 axial slices from 70 patients, without retraining or fine-tuning. A controlled ablation study comparing Nodule-only with Nodule + Anatomical Context was performed. **Results:** The anatomical-contextual scheme showed consistent improvements across all five architectures, with mean absolute gains of +0.078 in Precision, +0.122 in Recall, +0.101 in F1-score, +0.079 in mAP@50, and +0.190 in mAP@50–95. During internal validation, YOLOv10m achieved the highest Precision (0.979) and mAP@50 (0.987), as well as the shortest inference time (9.25 ms), whereas YOLOv11m achieved the highest Recall (0.971) and F1-score (0.962). During external evaluation, YOLOv10m retained the highest Precision (0.924) and mAP@50 (0.956), while YOLOv8m achieved the highest Recall (0.930) and mAP@50–95 (0.845); both models achieved an F1-score of 0.922. **Conclusions:** These findings provide experimental evidence that anatomical-contextual learning improves pulmonary nodule detection while maintaining competitive performance across datasets, supporting its potential as an anatomically informed and interpretable strategy for AI-assisted thoracic CT analysis.

## 1. Introduction

**Figure 1:**
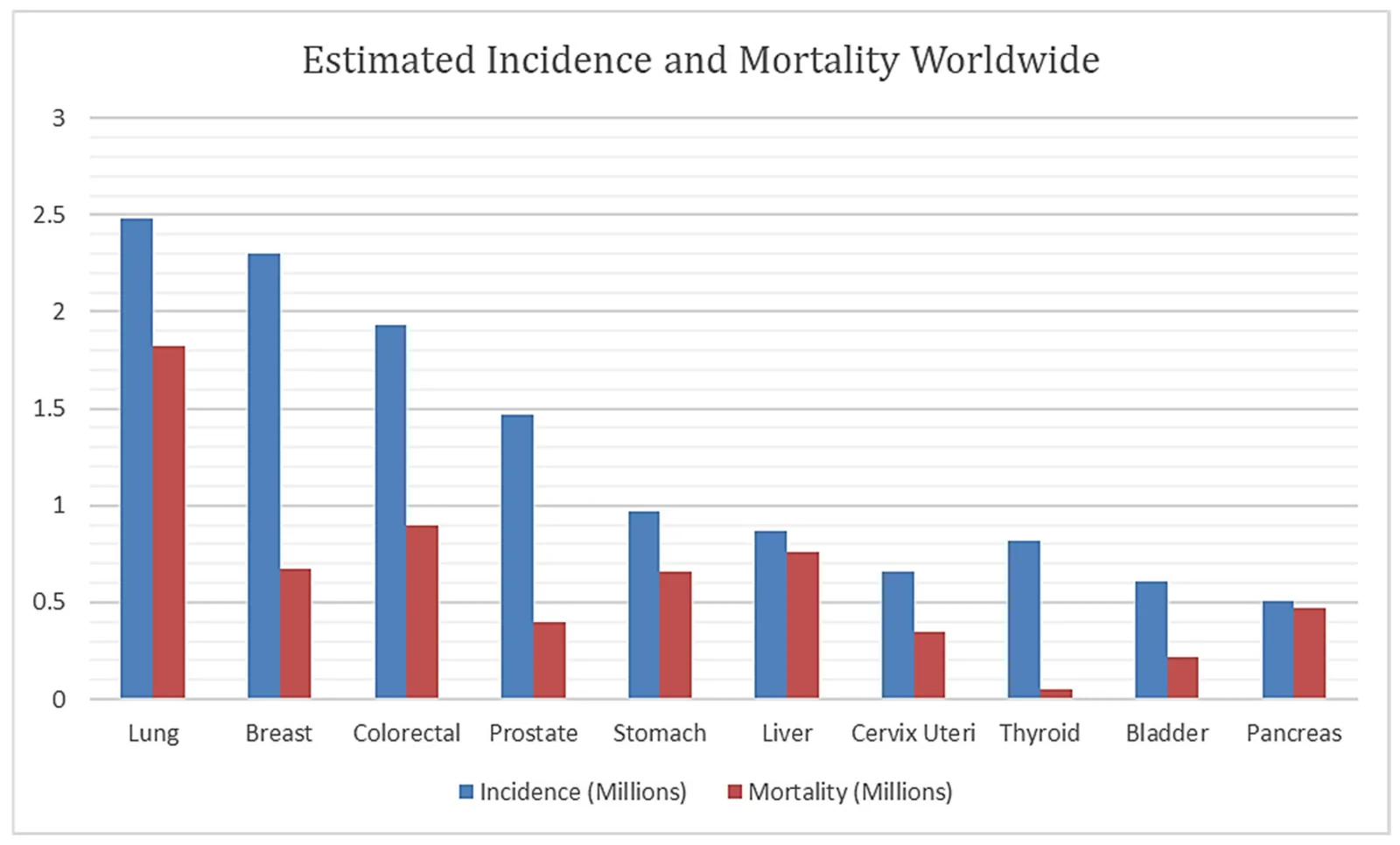
Global incidence and mortality estimates for major cancer types worldwide based on GLOBOCAN 2022 statistics, highlighting lung cancer as the leading cause of cancer-related mortality.

Lung cancer remains a major global public health challenge and is the leading cause of cancer-related mortality worldwide [1]. In 2020, approximately 2 million new cases and 1.8 million deaths were reported, underscoring the aggressive nature of the disease and the need for more effective early-detection strategies. Mexico faces a similarly substantial burden: 7811 new lung cancer cases and 6733 deaths were reported in the country [2]. The global incidence and mortality estimates for major cancer types are summarized in Figure 1.

Lung cancer arises from uncontrolled proliferation of abnormal pulmonary cells, which may progressively invade adjacent tissues and metastasize to distant organs. Prognosis deteriorates substantially once the disease reaches advanced stages, when therapeutic options become more limited and less effective [3]. This clinical course emphasizes the importance of diagnostic tools capable of identifying pulmonary nodules at earlier stages, when intervention may have a greater impact on patient outcomes.

### 1.1. Pulmonary Nodules and Their Relationship to Lung Cancer

A pulmonary nodule is defined as a rounded or oval pulmonary opacity measuring 3 cm or less and is most commonly detected on computed tomography (CT) [4,5]. Pulmonary nodules may represent benign, inflammatory, or transient processes, but they may also constitute an early manifestation of malignancy. Compared with conventional radiography, CT offers substantially greater sensitivity for nodule detection, identifying nodules in up to 17% of examinations versus approximately 2.1% of chest radiographs [4,6]. This improved sensitivity enables the detection of subclinical lesions at stages when clinical intervention may have a greater prognostic impact [7].

The clinical relevance of pulmonary nodules lies in their association with the subsequent risk of lung cancer [5]. In a cohort of more than 627,000 Medicare beneficiaries, the incidence of cancer after the initial detection of a nodule was 3.2% during the first year and increased to 4.7% in the second year [8]. In addition, screening studies have shown that up to 30% of carcinomas diagnosed at a localized stage had a documented nodule in previous imaging studies [9]. These findings highlight pulmonary nodules as clinically important radiological findings for which timely detection and appropriate follow-up may alter the subsequent diagnostic pathway.

However, the presence of a pulmonary nodule does not necessarily indicate malignancy. Its risk depends on morphological, volumetric, topographic, and clinical features [10,11]. For example, new lesions larger than 200 mm^3^ may be associated with malignancy rates approaching 11%, whereas nodules with typical perifissural morphology or specific subsolid patterns may present considerably lower risks [7]. This variability has driven the development of standardized stratification systems such as C-Lung-RADS, which integrates demographic, clinical, and radiological variables, achieving AUC values above 0.91 and outperforming approaches based solely on nodule size or density [12].

In this context, systematic pulmonary nodule detection is relevant not only to formal lung cancer screening programs but also to the assessment of incidental findings [13]. More than 50% of lung cancer cases may be diagnosed outside dedicated screening programs, emphasizing the need for automated tools capable of detecting, contextualizing, and prioritizing nodules across heterogeneous clinical settings [4,14]. Improving detection and follow-up strategies is therefore essential for strengthening early diagnosis and reducing lung cancer-related mortality [1,15,16].

### 1.2. AI-Assisted Diagnosis of Pulmonary Nodules

Artificial intelligence (AI) has become increasingly important for the automated analysis of computed tomography (CT), the primary imaging modality for detecting pulmonary lesions [17,18]. Low-dose CT (LDCT) screening has been associated with reductions in lung cancer mortality of 20–43% by enabling the detection of subclinical lesions before symptom onset [17,18,19]. However, the increased sensitivity of CT also increases the radiologists’ interpretation workload and creates a need for systems capable of distinguishing clinically relevant nodules from normal anatomical structures and benign findings.

Deep learning models, particularly convolutional neural networks, have demonstrated high performance in thoracic detection, classification, and segmentation tasks. AI-based computer-aided diagnosis (CAD) systems can achieve AUC≥0.95 and reduce false positives to fewer than 0.1 false positives per study [20], highlighting their potential to improve diagnostic sensitivity and support clinical decision-making. In this context, YOLO (You Only Look Once) detectors are particularly attractive for this task because their one-stage architecture enables computationally efficient real-time detection and multiscale feature extraction [21,22].

Nevertheless, pulmonary nodule detection should not be addressed solely as a local lesion-recognition problem. The visual similarity between nodules, vessels, mediastinal regions, cardiac structures, and diaphragmatic areas may generate ambiguity and false positives, particularly in small or low-contrast nodules [23]. Therefore, this study proposes an anatomical-contextual framework based on YOLOv8–YOLOv12, in which supporting thoracic structures are incorporated as spatial references during training, while the pulmonary nodule is maintained as a single invariant pathological class.

This approach is intended to support the development of more accurate, efficient, and interpretable CAD systems in a clinically relevant setting: five-year survival may reach 82–95% when lung cancer is diagnosed at an early stage, compared with approximately 15% at advanced stages [24]. Integrating AI, CT, and anatomical-contextual learning may therefore contribute to improved early detection of one of the leading causes of cancer-related mortality worldwide [25].

### 1.3. State of the Art in Deep Learning Architectures

Early detection of pulmonary nodules in CT has driven the development of deep learning-based CAD systems [26], owing to the ability of convolutional neural networks (CNNs) to extract discriminative spatial patterns directly from medical images [27,28]. Several recent approaches have incorporated explainable architectures, Vision Transformers, deformable convolutions, Squeeze-and-Excitation mechanisms, 3D models, Faster R-CNN with Swin Transformer, multiscale segmentation, and volumetric radiological descriptors, with the aim of improving the detection of small lesions, reducing false positives, and strengthening the anatomical characterization of pulmonary nodules [3,29,30,31].

Despite these advances, the recent literature identifies persistent limitations related to methodological heterogeneity, dependence on public datasets, limited external validation, and restricted explicit incorporation of thoracic anatomical context during training [32]. Although current models can achieve high levels of sensitivity and Precision, many approaches remain focused on isolated nodule detection through regions of interest (ROI), without comprehensively modeling the spatial relationships among the lungs, heart, spinal cord, digestive structures, and anatomically complex regions.

In this scenario, YOLO-based detectors have become increasingly prominent because of their ability to perform real-time detection with a competitive balance among Precision, sensitivity, and computational efficiency. Recent studies have incorporated attention mechanisms and hybrid CNN–Transformer strategies to improve the recognition of small or low-contrast nodules; however, most approaches prioritize quantitative performance without explicitly modeling the broader anatomical context that may improve interpretability.

Therefore, the present work proposes a multi-organ anatomical-contextual framework based on YOLOv8–YOLOv12, in which the lungs, heart, spinal cord, and digestive region are integrated as spatial references during training, while the pulmonary nodule is preserved as a single invariant pathological class. This formulation aims to move beyond isolated local detection and guide deep learning toward a more structured, interpretable, and robust thoracic representation under interpatient anatomical variability.

To contextualize the proposed framework within recent developments in AI-assisted pulmonary nodule detection, Table 1 contrasts representative deep learning approaches in terms of dataset, imaging modality, model architecture, methodological focus, and reported performance.

### 1.4. Research Questions and Evaluation Objectives

This study aims to analyze the impact of anatomical-contextual learning on automatic pulmonary nodule detection using YOLO-family architectures applied to computed tomography (CT) images. In particular, this study evaluates whether incorporating supporting thoracic anatomical structures as spatial context produces quantifiable improvements in pulmonary nodule detection compared with a reference training strategy focused exclusively on the Nodule class.

To directly quantify the contribution of anatomical context, a controlled ablation study was incorporated in which two training schemes were compared: Nodule-only and Nodule + Anatomical Context. The dataset partition, input resolution, hyperparameter configuration, optimization strategy, and evaluation protocol were kept constant between both conditions. This experimental design makes it possible to isolate the effect of the supporting anatomical classes from differences attributable to model architecture or training conditions.

#### Research Questions

- Does the incorporation of supporting thoracic anatomical structures—including the lungs, heart, spinal cord, and digestive region—improve pulmonary nodule detection performance compared with an equivalent Nodule-only training strategy?
- How does the magnitude of the benefit provided by anatomical context vary across YOLOv8, YOLOv9, YOLOv10, YOLOv11, and YOLOv12 when comparing Nodule-only with Nodule + Anatomical Context under identical experimental conditions?
- How consistently do models trained on LUNG-PET-CT-DX perform when externally evaluated on LIDC-IDRI without retraining, fine-tuning, or transfer learning?

### 1.5. Novelty and Contributions

The main contribution of this work is a multi-organ anatomical-contextual framework for pulmonary nodule detection in computed tomography images using YOLOv8–YOLOv12 architectures. Unlike approaches focused exclusively on isolated lesion detection, the proposed methodology incorporates supporting thoracic anatomical structures as spatial references during training while preserving the pulmonary nodule as a single invariant pathological class. The main contributions of this study are as follows:

1. **Dynamic axial thoracic labeling strategy:** An annotation strategy based on the morphological subdivision of thoracic structures along the axial axis is proposed to reduce intra-class variability and promote more consistent anatomical representations during learning.
2. **Multi-organ anatomical-contextual representation:** The lungs, heart, spinal cord, and digestive region are incorporated as supporting anatomical structures that function as complementary spatial references during training.
3. **Invariant pulmonary nodule detection:** The pulmonary nodule is maintained as a single pathological class regardless of anatomical location to encourage recognition based on intrinsic lesion characteristics such as texture, density, morphology, borders, and local contrast.
4. **Controlled ablation study:** A direct comparison is performed between a Nodule-only training scheme and the proposed Nodule + Anatomical Context scheme while maintaining the same dataset partition, input resolution, hyperparameters, optimization strategy, and evaluation protocol. This comparison directly quantifies the contribution of the supporting anatomical structures to pulmonary nodule detection performance.
5. **Comparative evaluation of YOLOv8–YOLOv12:** Five generations of YOLO architectures are analyzed under a homogeneous experimental protocol using common performance metrics, both under the anatomical-contextual scheme and in the controlled ablation study.
6. **Validation with heterogeneous datasets:** LUNG-PET-CT-DX is used for training and internal validation, whereas LIDC-IDRI serves as an independent external evaluation dataset without retraining or fine-tuning, thereby enabling assessment of framework transferability under domain shift.

## 2. Materials and Methods

### 2.1. Proposed Anatomical-Contextual Framework

**Figure 2:**
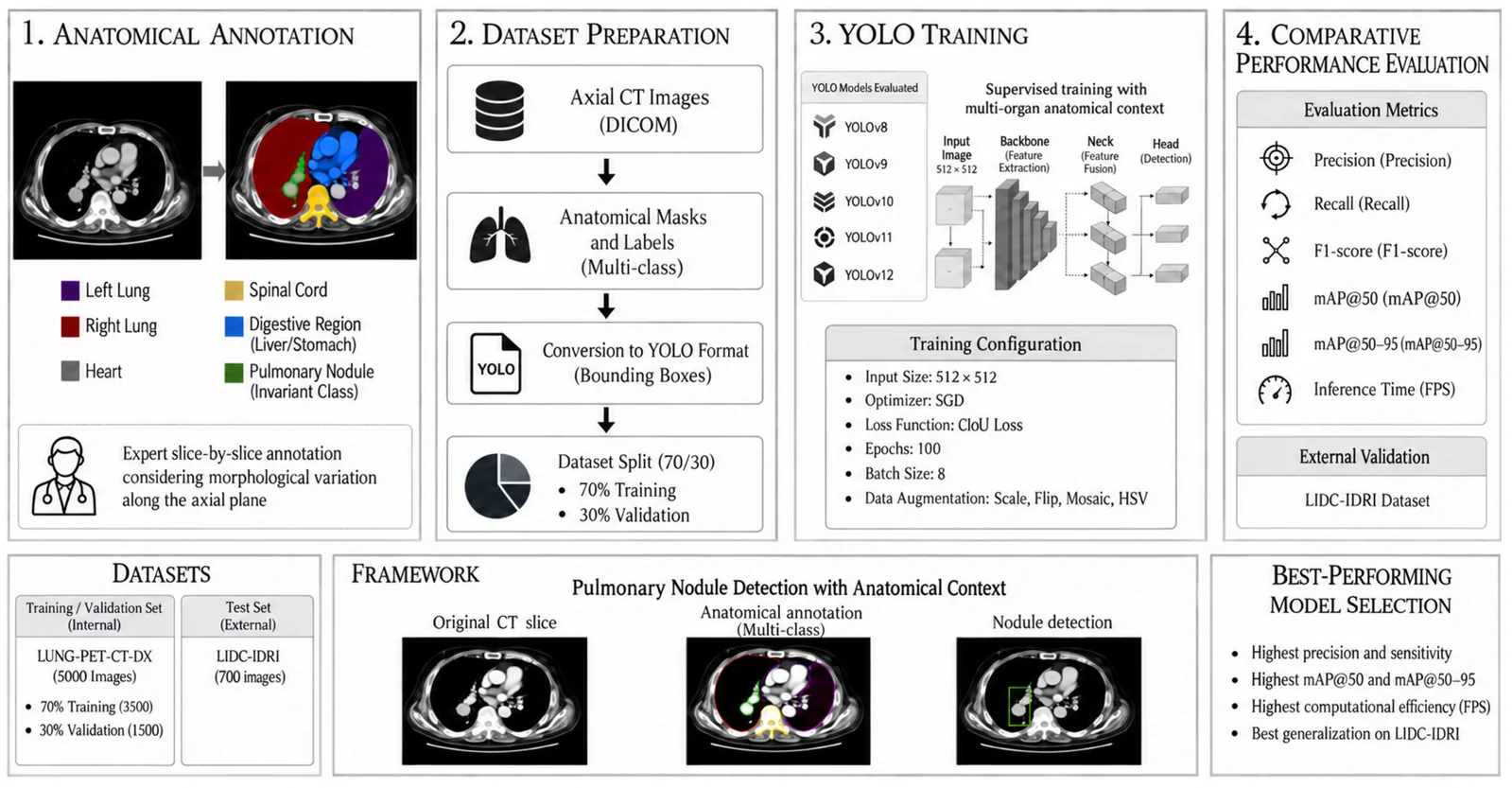
General workflow of the proposed multi-organ anatomical-contextual framework for pulmonary nodule detection in CT images. The scheme summarizes the stages of anatomical labeling, data preparation, training with YOLOv8–YOLOv12, and comparative performance evaluation through internal validation and external evaluation. The colors and the green square are used only for representative visualization, while the arrows indicate the sequential flow between the different stages of the proposed framework.

The proposed framework consists of four main stages: (1) anatomical-contextual labeling of thoracic structures, (2) dataset preparation and organization, (3) supervised training using YOLOv8–YOLOv12 architectures, and (4) comparative performance evaluation in pulmonary nodule detection tasks. The overall workflow of the proposed framework is illustrated in Figure 2.

The labeling strategy incorporates relevant anatomical structures, including the lungs, heart, spinal cord, and digestive region, to provide additional spatial context during convolutional neural network training. Under this scheme, the pulmonary nodule is treated as an invariant pathological class, whereas the anatomical structures serve as complementary spatial references that support a structured representation of the thoracic scene.

The annotated images were subsequently used to train multiple YOLO architectures and assess the effect of anatomical-contextual learning on automatic pulmonary nodule detection in CT images. Model performance was evaluated using Precision, Recall, F1-score, mAP@50, and mAP@50–95, together with external evaluation on LIDC-IDRI to assess cross-dataset generalization.

### 2.2. Dynamic Thoracic Labeling Strategy

The dynamic thoracic labeling strategy was designed to frame pulmonary nodule detection in CT as an anatomical-contextual interpretation task rather than solely as local lesion recognition. Under this approach, the pulmonary nodule remains the primary pathological target, while supporting thoracic structures—including the lungs, heart, spinal cord, and digestive region—are incorporated as spatial references during training.

This formulation enables the CNN to learn anatomically conditioned representations that capture not only the visual appearance of the nodule but also its spatial relationship with the surrounding thoracic anatomy. Such contextual information is particularly relevant in anatomically complex regions, where pulmonary vessels, mediastinal tissue, cardiac structures, or areas near the diaphragm may exhibit intensities, contours, and spatial patterns similar to those of small pulmonary lesions, thereby increasing the risk of false-positive detections.

By explicitly incorporating anatomical context, the detection process is extended beyond purely local image descriptors toward spatially guided contextual learning. This strategy encourages the extraction of anatomically coherent features and reduces reliance on isolated visual patterns that may resemble normal thoracic structures.

**Figure 3:**
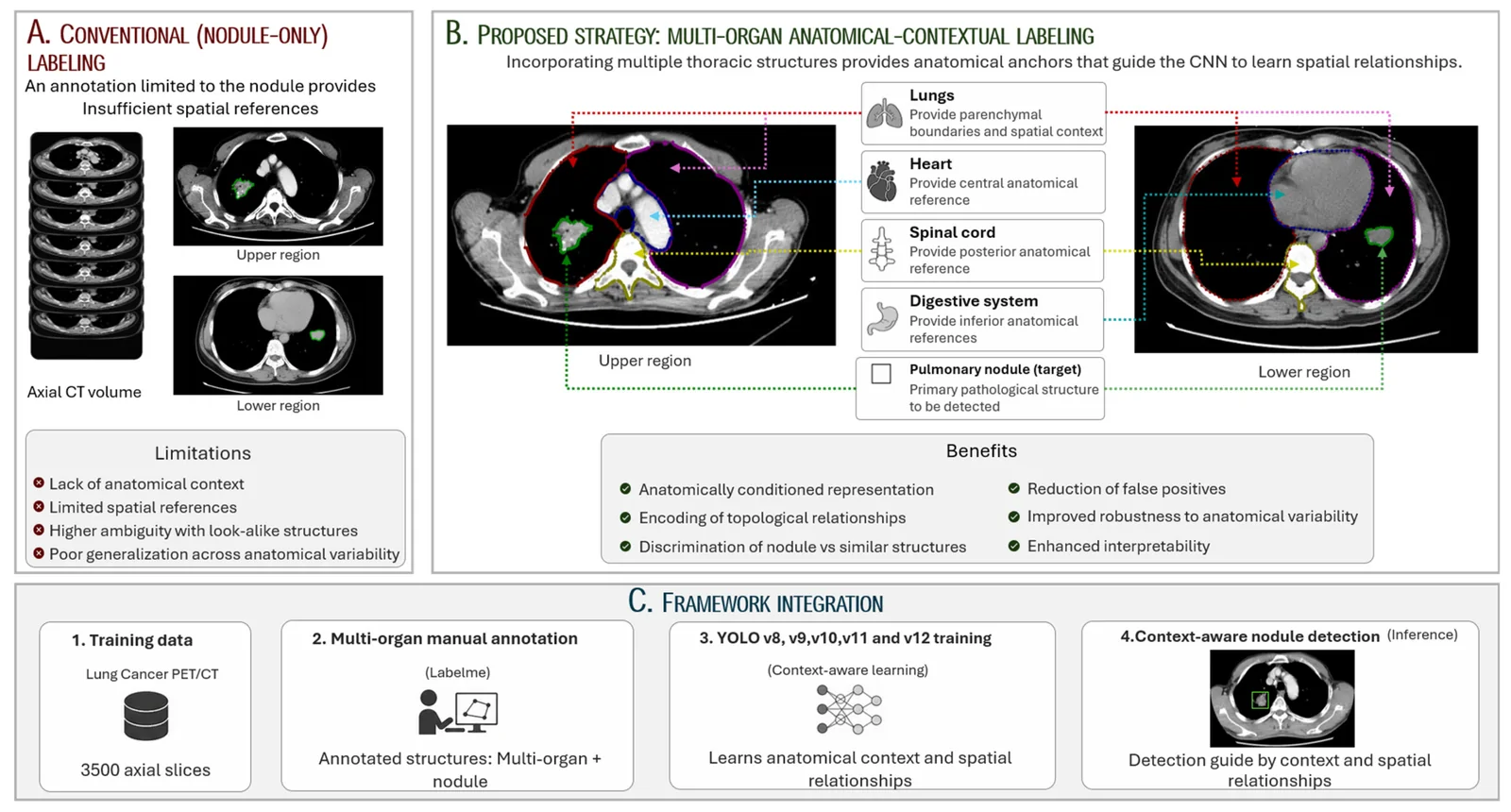
Dynamic thoracic labeling strategy: multi-organ anatomical-contextual labeling. The scheme incorporates supporting anatomical structures as auxiliary spatial references to induce anatomically conditioned convolutional learning. The green square is used as a representative marker for the pulmonary nodule target.

As shown in Figure 3, each axial slice is represented as a structured anatomical scene. The supporting structures provide spatial organization and inter-organ context, while the nodule remains a single pathological class. This scheme allows supervised learning to incorporate both local lesion features and broader anatomical relationships, resulting in a more structured and interpretable thoracic representation.

**Figure 4:**
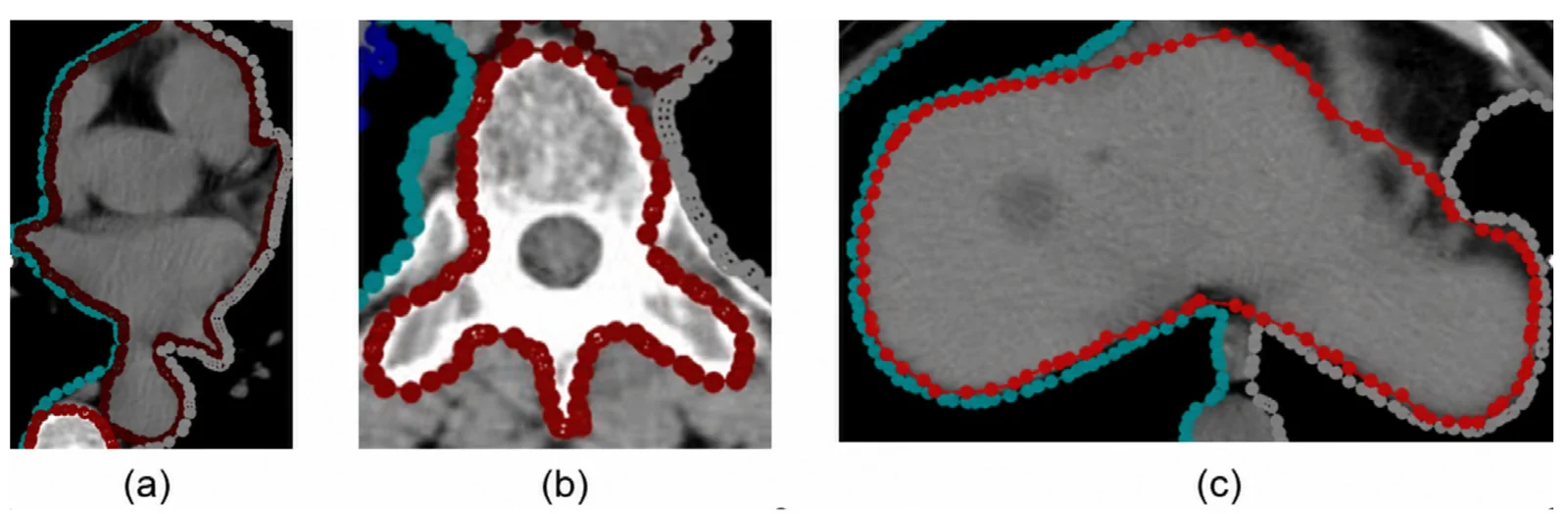
Examples of dynamically labeled anatomical structures in computed tomography (CT) images used as context for the object detection model. (**a**) shows different morphological representations of the heart. (**b**) presents an axial slice with the spinal cord structure (Spinal_cord class) delineated. (**c**) presents an axial slice highlighting a structure categorized as part of the digestive region. The different colors are used only for representative visualization of the manually delineated anatomical structures.

To implement this strategy, the eight supporting anatomical classes were manually labeled on 5000 CT slices from the LUNG-PET-CT-DX dataset using LabelMe (version 5.8.3). The original expert-provided tumor annotations were used as the clinical and topographic reference for the Nodule class, whereas the study-specific anatomical structures were manually delineated according to the proposed anatomical-contextual protocol. Polygonal annotations were subsequently converted into enclosing bounding boxes and transformed into the standardized YOLO representation, as detailed in Section 2.6. Representative examples of the anatomical labeling process are presented in Figure 4.

### 2.3. Morphological Classification of Support Structures

**Figure 5:**
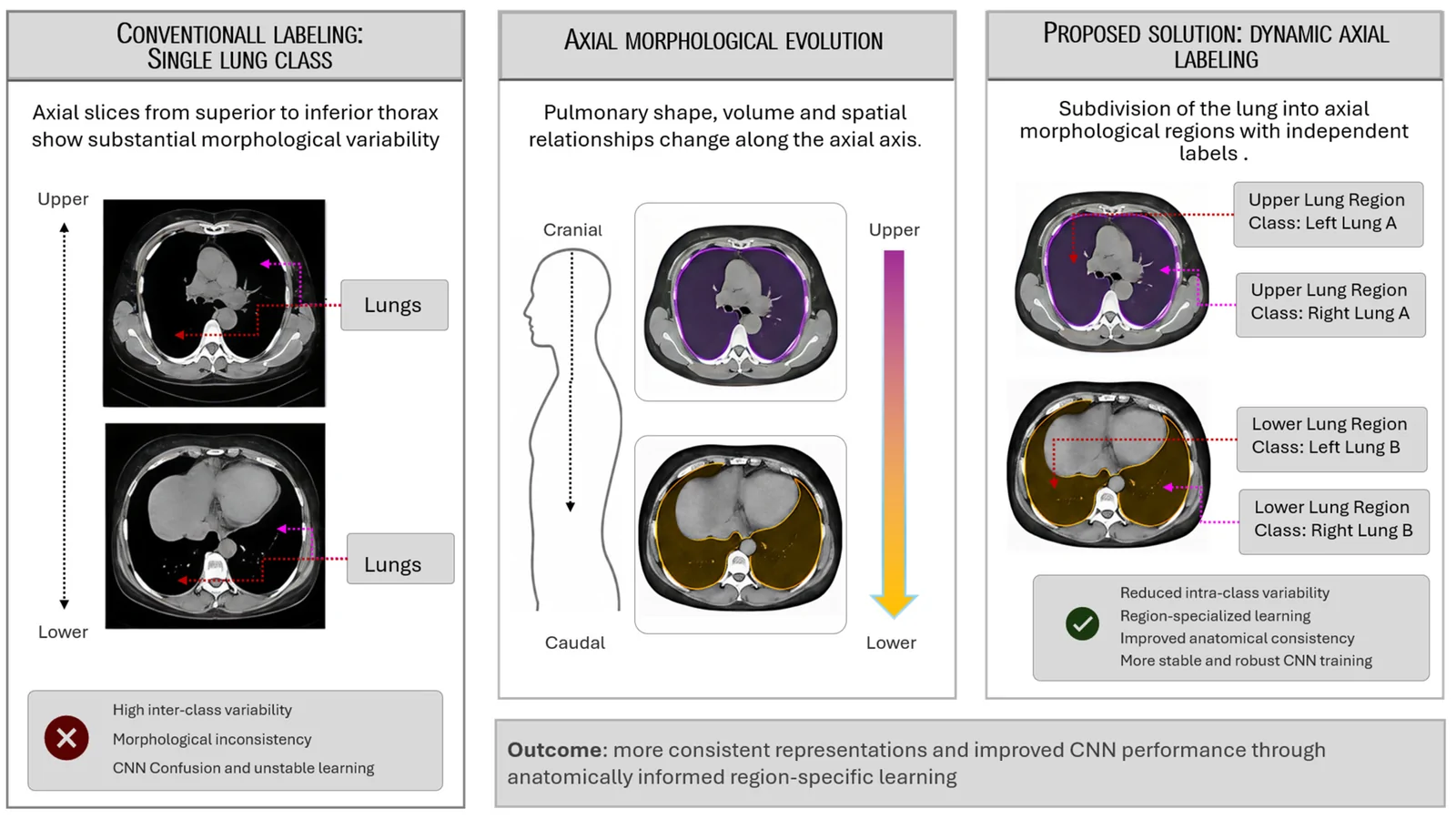
Conceptual representation of the axial morphological subdivision applied to pulmonary support structures. The scheme illustrates the progressive anatomical transition between superior and inferior thoracic regions used to reduce intra-class morphological variability during CNN training.

The supporting anatomical structures in thoracic CT exhibit substantial morphological variation along the axial axis. To reduce intra-class heterogeneity and improve reproducibility, the lungs and heart were subdivided into anatomically defined regional configurations using explicit landmarks rather than fixed percentages of the scan or purely subjective slice position. The conceptual representation of this axial morphological subdivision is illustrated in Figure 5.

For the pulmonary regions, the tracheal carina was used as the primary anatomical landmark. Lung A corresponded to the superior/apical pulmonary region extending from the lung apices to the axial level of the tracheal bifurcation. Lung B corresponded to the inferior/basal pulmonary region extending from the first slice immediately caudal to the carina to the visualization of the diaphragmatic dome. This rule was applied independently to the left and right lungs, generating the classes Left Lung A, Left Lung B, Right Lung A, and Right Lung B.

For the cardiac regions, the transition between Heart A and Heart B was defined according to reproducible morphological landmarks rather than ordinal slice position. Heart A corresponded to the superior cardiac configuration, including axial levels characterized by the presence of the great vessels, atrial structures, and the superior atrioventricular region. The transition to Heart B was identified by the progressive predominance of ventricular anatomy, including the interventricular septum and ventricular mass, extending inferiorly toward the cardiac apex.

**Figure 6:**
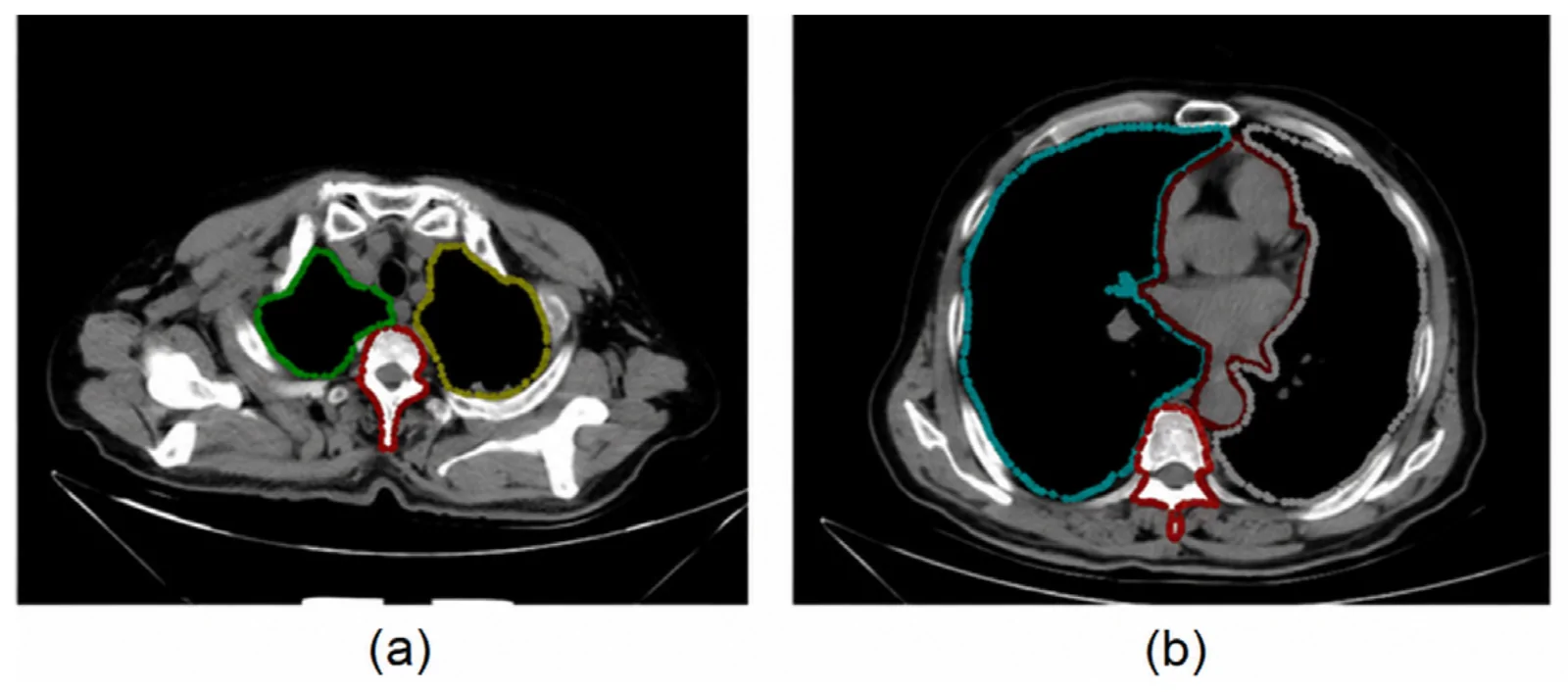
Representative examples of dynamic axial labeling in thoracic CT. The pulmonary regions correspond to (**a**) superior and (**b**) inferior thoracic levels, illustrating the morphological variation of the lungs along the axial axis and the rationale for representing these regions as separate classes. The different colors are used only for representative visualization of the labeled pulmonary regions.

In anatomically transitional cases, annotators were permitted to inspect adjacent superior and inferior slices to preserve volumetric anatomical continuity and avoid isolated-slice misclassification. Ambiguous assignments were subsequently resolved through the quality-control procedure described in Section 2.6. Unlike the supporting structures, the pulmonary nodule remained a single invariant pathological class regardless of anatomical level, thereby avoiding location-dependent pathological subclasses. Representative examples of the superior and inferior pulmonary regions are shown in Figure 6.

### 2.4. Lung Delimitation and Invariant Nodule Detection

Within the proposed framework, lung parenchyma representation was designed to distinguish contextual anatomy from the pathological representation of the pulmonary nodule. Supporting structures are modeled as axially conditioned anatomical classes, whereas the nodule is preserved as a single invariant pathological class regardless of its location within the thoracic cavity.

As described in Section 2.3, pulmonary structures undergo progressive morphological changes along the axial axis. Their subdivision into anatomically defined regions therefore reduces intra-class heterogeneity and supports more consistent feature learning. Applying the same subdivision principle to the pulmonary nodule, however, could create an artificial dependency between lesion identity and anatomical location, introducing undesirable spatial biases during training.

Maintaining a single Nodule class reduces the risk that the CNN will associate specific pulmonary regions with a higher probability of lesion occurrence. Subdividing nodules according to anatomical location could encourage the model to learn dataset-specific spatial correlations rather than lesion-specific characteristics. Consequently, all nodules share the same semantic representation during annotation and supervised training.

In contrast, the lungs and heart are represented using regional classes to capture their axial morphological variability. This separation between dynamic anatomy and invariant pathology allows the supporting structures to provide organized spatial context while nodule detection remains primarily driven by lesion-specific properties such as texture, density, borders, morphology, and local contrast.

**Figure 7:**
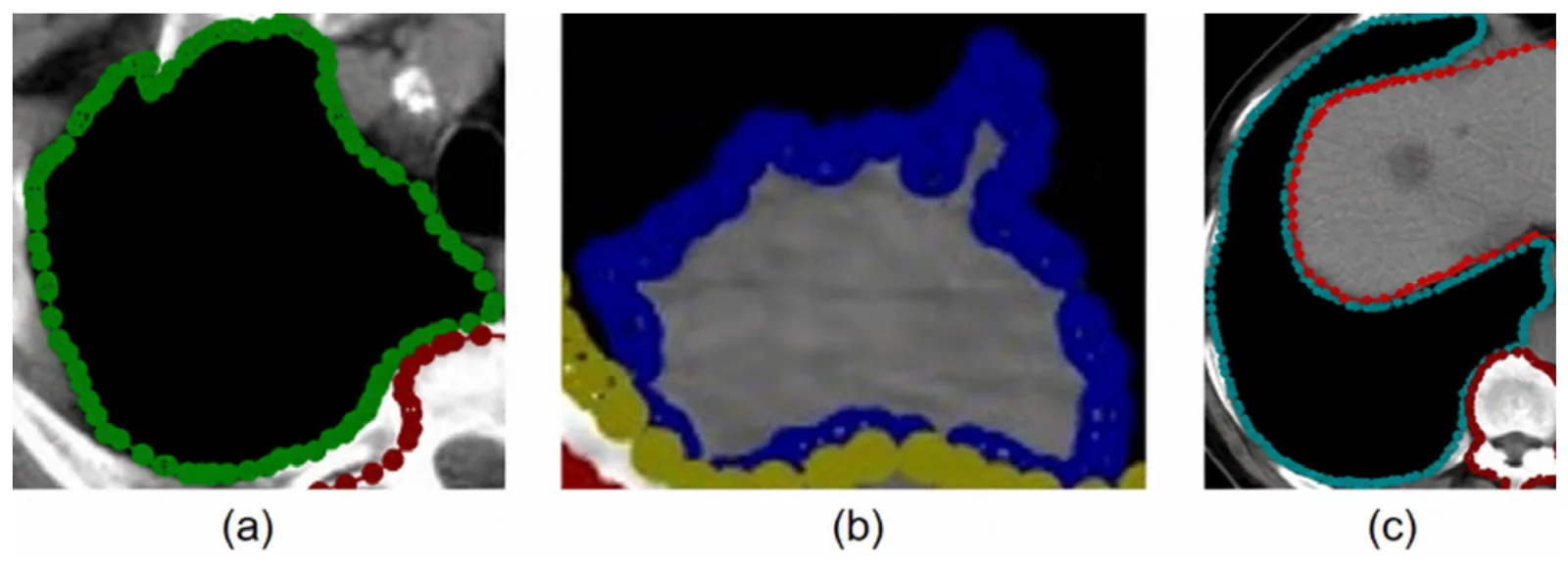
Anatomical-contextual and pulmonary nodule labeling. (**a**,**c**) illustrate the labeling of surrounding anatomical structures, whose purpose is to provide the model with structured anatomical context within each axial slice. In contrast, (**b**) details the direct delimitation of the lesion. This approach provides the network with the topographic context required to isolate and detect the nodule with greater Precision. The different colors are used only for representative visualization of the manually delineated regions.

As shown in Figure 7, the surrounding anatomical regions provide topographic references for interpreting the thoracic scene, whereas the nodule is delineated consistently regardless of its location. This formulation supports a hierarchical anatomical–pathological representation, reduces spatial bias, and may improve the ability of the model to generalize pulmonary nodule detection across CT images.

### 2.5. Dataset Selection and Cross-Dataset Evaluation Strategy

Generalization remains a major challenge for deep learning systems in medical imaging. In CT-based pulmonary nodule detection, model performance may be affected by differences in acquisition protocols, image reconstruction, and interpatient anatomy. Consequently, models that perform well on the training distribution may show substantial performance degradation when evaluated on external datasets.

To assess the generalization of YOLOv8–YOLOv12 under the anatomical-contextual framework described in Section 2.2, Section 2.3 and Section 2.4, an inter-dataset training and evaluation strategy was implemented using two independent datasets:

- **LUNG-PET-CT-DX** [38]: used for training and internal validation.
- **LIDC-IDRI** [39]: used exclusively for independent external evaluation.

The LUNG-PET-CT-DX dataset, available through The Cancer Imaging Archive (TCIA), contains thoracic CT and PET-CT studies oriented toward lung cancer diagnosis. For this research, 5000 axial CT slices from 320 patients were selected using a controlled representation criterion aimed at maintaining adequate coverage of the supporting anatomical structures and pathological target without enforcing exact numerical class balance.

The multi-organ anatomical-contextual learning scheme described above was implemented using this dataset and included the following:

- Incorporation of lungs, heart, spinal cord, and digestive region as supporting anatomical structures;
- Definition of the pulmonary nodule as the main pathological target;
- Axial subdivision of pulmonary regions into superior and inferior lung and heart configurations;
- Manual annotation using LabelMe under the proposed anatomical-contextual scheme.

The experimental strategy employed hold-out validation with a 70/30 distribution for training and internal validation. The general characteristics of the training and validation dataset are summarized in Table 2.

For external evaluation, an independent subset of LIDC-IDRI, a widely used reference dataset for pulmonary nodule research, was selected. A total of 700 axial slices from 70 patients were included, with 10 representative slices selected per patient. This external dataset was used to evaluate the following:

- Spatial generalization of the proposed framework;
- Robustness against tomographic distributions not observed during training;
- Stability of the learned anatomical representations;
- Performance of YOLOv8–YOLOv12 under interpatient anatomical variability.

The LIDC-IDRI dataset provides radiologist-based pulmonary nodule annotations through associated XML metadata files generated during a two-phase reading process involving four experienced thoracic radiologists. In this study, the original metadata-integrated annotations were used as the reference for the Nodule class during external evaluation. In addition, the selected external CT slices were manually annotated for the eight auxiliary anatomical-context classes, Left Lung A, Left Lung B, Right Lung A, Right Lung B, Heart A, Heart B, Spinal Cord, and Digestive Region, following the same annotation protocol, axial morphological criteria, and two-stage quality-control procedure described in Section 2.6. These study-specific anatomical annotations were used exclusively as reference labels during external evaluation; neither the LIDC-IDRI images nor their study-specific anatomical annotations were used for training, internal validation, fine-tuning, or domain adaptation.

The central experimental hypothesis was that axially conditioned anatomical-contextual learning could produce representations that are more robust to dataset-specific variation. Accordingly, the external evaluation was designed not only to measure detection performance but also to determine whether explicit multi-organ anatomical context supports cross-dataset generalization in automatic pulmonary nodule detection. The main characteristics of the LIDC-IDRI dataset used for independent external evaluation are summarized in Table 3.

**Figure 8:**
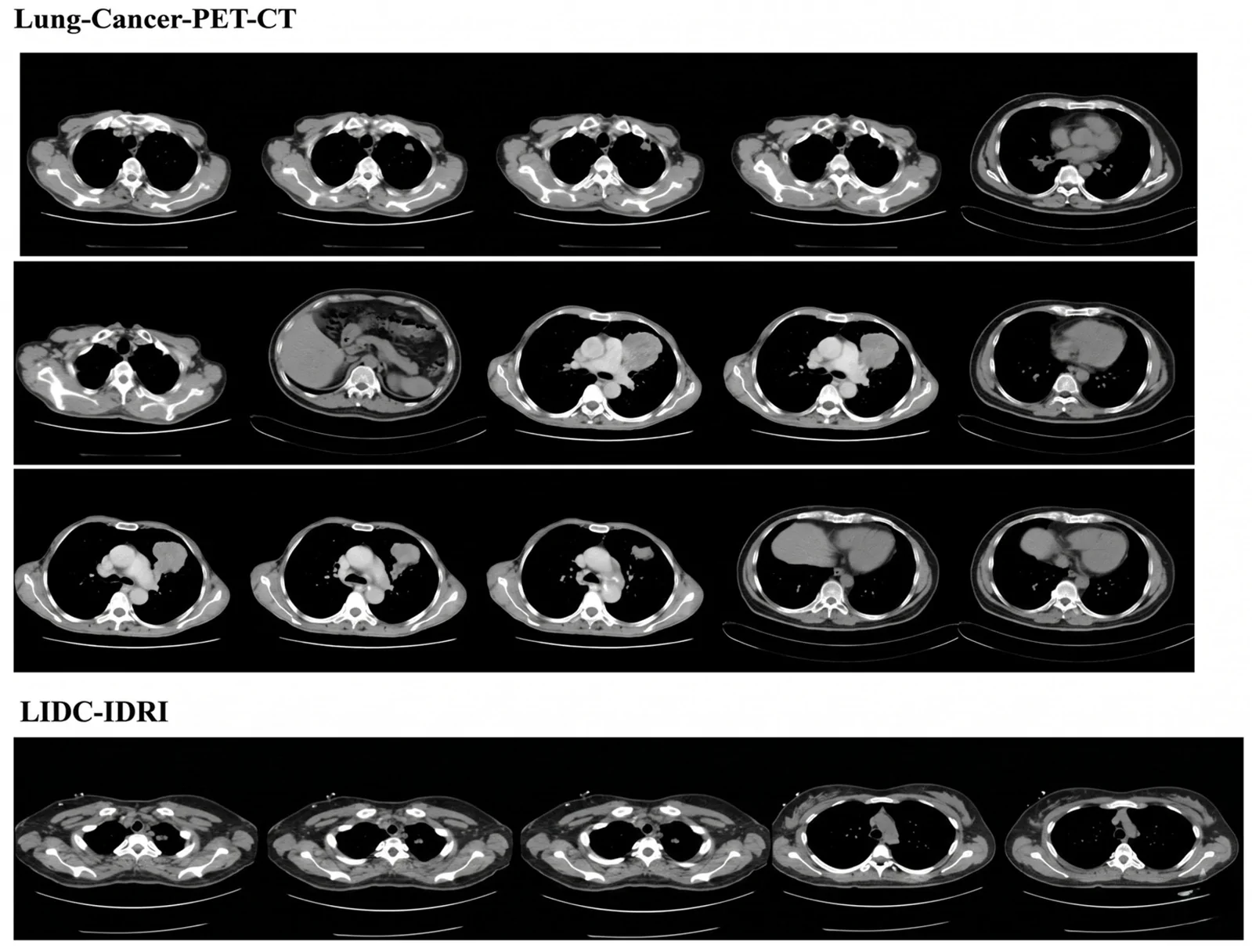
Example images from the LUNG-PET-CT-DX and LIDC-IDRI datasets used for training, internal validation, and external evaluation. The datasets include CT slices with diverse anatomical structures and pathological variations relevant to pulmonary nodule detection.

Figure 8 shows examples of the CT images used in the training and evaluation implemented in this study. The framework was trained using LUNG-PET-CT-DX under the proposed anatomical representation scheme, whereas spatial generalization capability was evaluated through inference on independent studies from the LIDC-IDRI dataset.

#### Image Preparation and Input Representation

For LUNG-PET-CT-DX, the selected CT images were originally available in DICOM format and were converted to PNG using DICOM2PNG (version 2023.12.31) before annotation and model training. No study-specific manual transformation of Hounsfield Unit (HU) values, custom HU clipping, or multichannel window encoding was implemented. The pulmonary window (W = 1400 HU, L = −700 HU) and mediastinal window (W = 350 HU, L = 40 HU) reported for LUNG-PET-CT-DX in Table 2 correspond to documented imaging characteristics of the source dataset and were not independently encoded into separate network channels during the present experiments.

The resulting PNG images preserved the grayscale tomographic appearance and were subsequently handled by the standard Ultralytics (version 8.3.203) image-loading pipeline as three-channel network inputs. No distinct CT windows or additional spectral information were assigned to individual channels; therefore, the three-channel representation was used only to satisfy the input requirements of the evaluated YOLO architectures. No encoding such as pulmonary-window-to-R, mediastinal-window-to-G, or alternative HU-window-to-B was used.

The original CT images had a spatial resolution of 512 × 512 pixels, and the experimental input size was likewise configured as imgsz = 512. No additional manual downsampling was performed before training. Numerical normalization and spatial conditioning required for network input were handled by the standard Ultralytics preprocessing pipeline.

Standard Ultralytics data augmentation was maintained during training, including HSV-based transformations. Because the CT images were essentially achromatic, hue and saturation perturbations had limited effect, whereas modifications associated with the value component primarily introduced intensity or brightness variations. Accordingly, HSV augmentation was treated as part of the standard training augmentation pipeline rather than as a source of clinically meaningful color information.

### 2.6. Annotation Protocol and Quality Control

The annotations used in this study had two distinct sources. Pulmonary nodule localization was based on the expert radiological annotations originally provided with the public datasets. For LUNG-PET-CT-DX, the original tumor-location annotations available in the associated XML metadata were generated under the dataset’s radiological annotation protocol involving five academic thoracic radiologists and were used in the present study as the clinical and topographic reference for the Nodule class. Two radiologists had more than 15 years of experience and three had more than 5 years of experience. One radiologist annotated each subject, while the remaining radiologists participated in verification and review of the annotation records. These original expert annotations were not redefined by the study annotators.

The eight anatomical-context classes—Left Lung A, Left Lung B, Right Lung A, Right Lung B, Heart A, Heart B, Spinal Cord, and Digestive Region—were generated specifically for this study. Manual anatomical labeling of the selected CT slices was performed by a multidisciplinary team of ten annotators, comprising seven undergraduate students completing social-service training, two faculty researchers with doctoral training, and one master’s student. All annotators followed the same predefined anatomical labeling protocol and the morphological criteria described in Section 2.2, Section 2.3 and Section 2.4.

Annotations were created in LabelMe using polygon-based delineation. The original expert-provided lesion annotations served as the clinical reference for pulmonary nodule localization, while the study-specific anatomical structures were manually delineated as polygons. For model training and evaluation, each polygonal annotation was converted into its enclosing bounding box and subsequently transformed into the YOLO coordinate representation $x_{center},y_{center},\;width,\;height$. This procedure preserved the anatomical extent defined during manual delineation while providing a standardized object-detection representation compatible with YOLOv8–YOLOv12.

The eight anatomical classes were incorporated exclusively as auxiliary contextual references and were not intended as independent diagnostic targets or clinically validated organ-segmentation outputs. The principal pathological endpoint remained the invariant Nodule class.

Annotation quality was controlled through a multistage review process. The entire annotation procedure was conducted under the supervision of I.P.-B., a radiologist affiliated with a tertiary oncology center. After the initial labeling stage, all anatomical delimitations and A/B class assignments underwent a complete second review by L.M.-G., a biomedical specialist, who corrected labeling inconsistencies and standardized anatomical boundaries. The revised annotations then underwent final radiological review and validation by I.P.-B. For anatomically ambiguous transition slices, adjacent superior and inferior CT slices were reviewed to support consistent class assignment.

Because the annotation workflow was based on a unified clinical reference and sequential expert quality control rather than independent blinded duplicate labeling of the same lesion, no formal interobserver or intraobserver agreement coefficient was calculated. Accordingly, no consensus threshold or mathematical fusion operation was required for lesion localization.

### 2.7. YOLO-Based Architectures for Anatomical-Contextual Learning

The proposed framework treats pulmonary nodule detection as an anatomical-contextual interpretation task in which the lesion is analyzed together with supporting thoracic structures. Accordingly, the detection architecture must capture both local nodule characteristics and broader spatial relationships within anatomically complex CT images.

YOLO-family architectures were selected because of their one-stage detection design, computational efficiency, and ability to extract hierarchical multiscale features [40]. Unlike approaches based on isolated regions of interest, YOLO processes the complete image during inference, preserving global anatomical context and supporting scene-level representations. This capability is particularly relevant for small or low-contrast nodules and for lesions located near pulmonary vessels, the mediastinum, the heart, or the diaphragm [32].

The applicability of YOLO to this task has been demonstrated by recent models such as ELCT-YOLO [23], YOLO-ED [33], WTAM-YOLO [22] and YOLOv10-based variants [15], all of which target pulmonary nodule or tumor detection in CT. Other approaches incorporating attention mechanisms, optimized loss functions, and improved feature propagation have also sought to increase sensitivity to small lesions and complex morphologies [34].

Architecturally, YOLO combines a backbone, neck, and detection head to support hierarchical feature extraction, multiscale aggregation, and efficient object localization. Comparing YOLOv8–YOLOv12 therefore enables evaluation of how different architectural refinements interact with the proposed multi-organ anatomical-contextual learning strategy.

#### Comparative Evaluation of YOLOv8–YOLOv12

YOLOv8–YOLOv12 architectures belong to the family of one-stage object detectors and share a general structure based on a backbone, neck, and detection head. This formulation enables the extraction of hierarchical representations, the fusion of multiscale features, and the simultaneous regression of bounding boxes and object classification in a single pass. Under the proposed anatomical-contextual framework, this common foundation makes it possible to compare how different architectural refinements respond to the incorporation of supporting thoracic structures during pulmonary nodule detection in CT [41].

**YOLOv8**: This architecture combines a CSPNet-based backbone, an FPN+PAN neck, and a decoupled detection head with an anchor-free formulation [42]. This configuration simplifies object assignment, supports more stable convergence, and strengthens the localization of small objects through feature fusion at different scales. Within the proposed framework, these properties are relevant for processing the complete thoracic scene and discriminating pulmonary nodules from anatomical structures with partially similar appearance, such as pulmonary vessels, the mediastinum, cardiac regions, and diaphragmatic areas [43].

**YOLOv9**: This architecture incorporates GELAN (Generalized Efficient Layer Aggregation Network) and PGI (Programmable Gradient Information), which are designed to improve gradient propagation and preserve discriminative information during training [44]. GELAN promotes efficient reuse of deep features and multiscale aggregation, whereas PGI reduces the loss of spatial information in deep layers. These properties are particularly relevant in the anatomical-contextual approach, where the model must integrate multi-organ references and subtle nodular patterns distributed across anatomically variable thoracic regions.

**YOLOv10**: This architecture introduces an NMS-free training and inference strategy based on dual assignment [45]. The one-to-many branch provides dense supervision during training, strengthening the learning of complex patterns, whereas the one-to-one branch generates direct predictions during inference, reducing dependence on post-processing through Non-Maximum Suppression. In addition, its scalable variants make it possible to adjust the balance between Precision and computational cost. In this study, these characteristics are relevant because they combine inference efficiency, training stability, and multiscale representation for the detection of small or low-contrast nodules.

**YOLOv11**: This architecture maintains the one-stage paradigm and incorporates refinements oriented toward parametric efficiency, gradient flow, and accelerated deployment. Its architecture integrates C3k2 blocks to optimize hierarchical extraction, CSP/C2PSA modules to improve the reuse of deep representations, and a PANet neck for multiscale aggregation [46,47]. These characteristics enable the integration of semantic and spatial information from multiple resolutions, preserving discriminative patterns associated with both the pulmonary nodule and the supporting anatomical structures. Its compatibility with ONNX, TensorRT, CoreML, and TFLite also supports its potential integration into real-time CAD processing systems [35].

**YOLOv12**: This architecture incorporates a design more oriented toward contextual modeling through a Residual-ELAN (R-ELAN) architecture and efficient attention mechanisms, including FlashAttention [48]. R-ELAN seeks to stabilize information flow in deep networks through residual connections and efficient feature aggregation, whereas FlashAttention reduces the memory overhead associated with attention blocks. Additionally, the use of 7 × 7 separable convolutions as a position perceiver enables the implicit encoding of positional information, which is useful for representing complex thoracic structures and spatial relationships among organs [49].

Overall, the comparison of YOLOv8–YOLOv12 enables the evaluation of how different architectural strategies (anchor-free detection, gradient preservation, NMS-free dual assignment, parametric efficiency, compatibility with accelerated deployment, and attention mechanisms) influence the ability of the anatomical-contextual framework to detect pulmonary nodules in CT within a multi-organ thoracic scene.

## 3. Experimental Configuration and Results

To test the central hypothesis of this study, YOLOv8, YOLOv9, YOLOv10, YOLOv11, and YOLOv12 were evaluated under a common experimental protocol to determine whether multi-organ anatomical context improves pulmonary nodule detection in computed tomography (CT). The analysis focused on both detection performance and the ability of the models to learn anatomically consistent spatial representations within the thoracic cavity.

**Figure 9:**
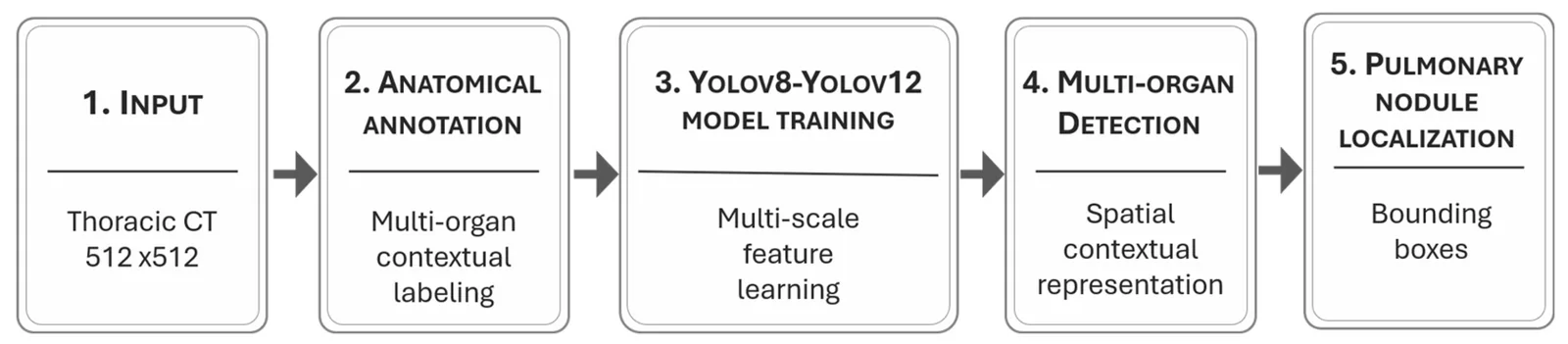
General diagram of the pulmonary nodule detection process in computed tomography (CT) images using YOLO-based convolutional neural networks (CNNs).

Figure 9 summarizes the experimental workflow. Starting from axial CT images, the proposed anatomical-contextual labeling strategy was used to incorporate supporting thoracic structures as spatial references during training. The five YOLO architectures were then trained under a common supervised protocol and evaluated for simultaneous detection of pulmonary nodules and supporting anatomical structures.

All experiments were conducted under controlled conditions using the same dataset partition, input resolution, hyperparameters, and evaluation criteria. This standardization reduced variability associated with the training protocol and enabled a more direct comparison among architectures and experimental conditions.

Performance was assessed using standard object-detection metrics, including Precision, Recall, mAP@50, and mAP@50–95, together with inference time, confusion matrices, and qualitative detection examples. The following subsections describe the experimental configuration, comparative performance of YOLOv8–YOLOv12, and the effect of anatomical-contextual learning on pulmonary nodule detection and cross-dataset generalization.

To perform an objective comparison among YOLOv8, YOLOv9, YOLOv10, YOLOv11, and YOLOv12 architectures, all models were trained under homogeneous experimental conditions. Standardizing the training configuration allows the observed performance differences to be attributed primarily to the architectural characteristics of each model rather than to variations in the optimization procedures.

### 3.1. Training Configuration

To ensure an objective and reproducible comparison among the YOLOv8–YOLOv12 architectures, a supervised training methodology was implemented under homogeneous experimental conditions. The experimental design was structured to evaluate both the intrinsic differences among architectures and the specific contribution of anatomical-contextual learning while maintaining consistent data preparation, training, and validation conditions.

**Figure 10:**
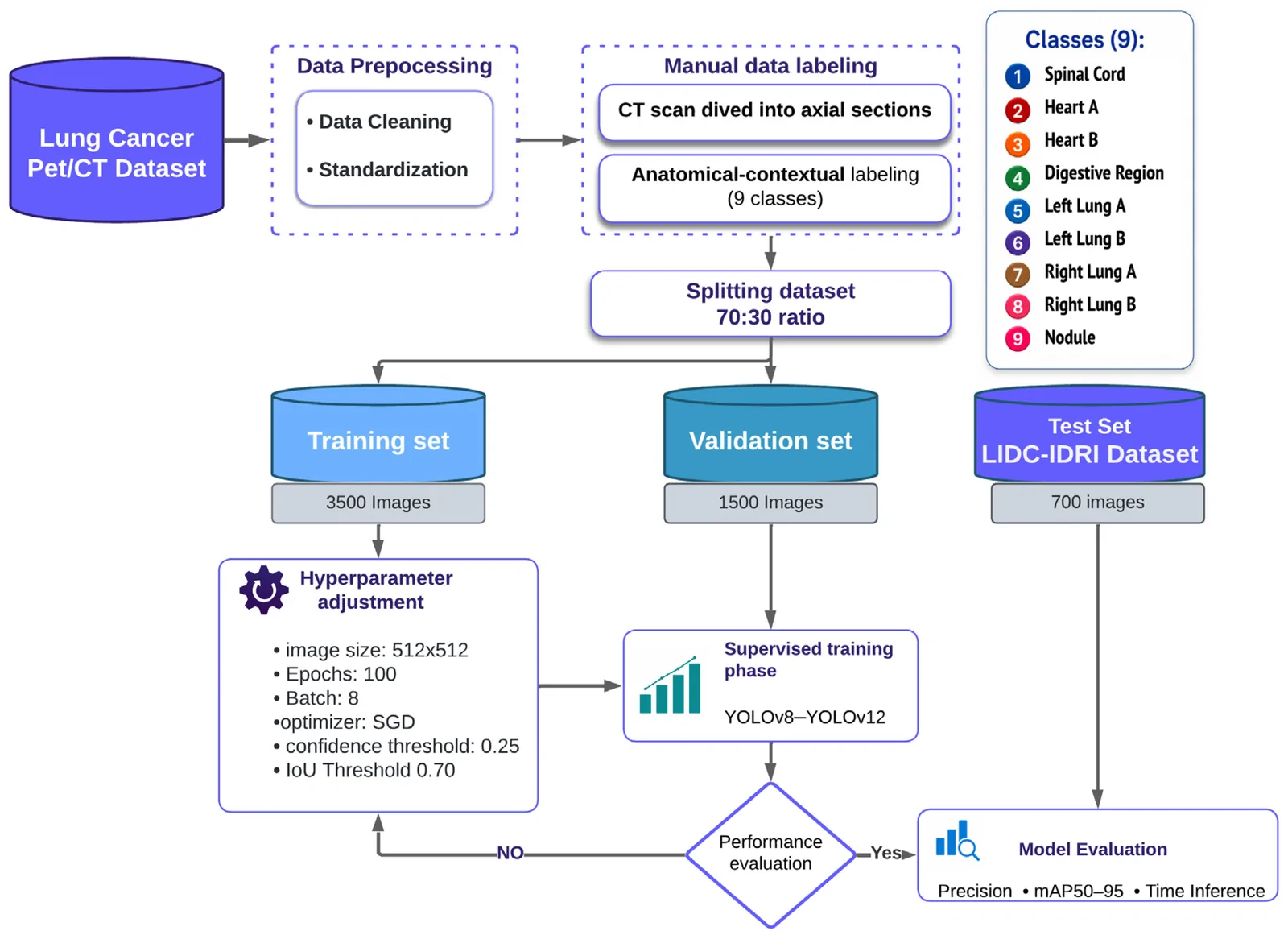
Experimental workflow used for training, internal validation, and external evaluation of the proposed anatomical-contextual framework.

Figure 10 summarizes the experimental methodology implemented in this study. Starting from CT images from the LUNG-PET-CT-DX dataset, axial slices were selected and prepared, including conversion of the selected DICOM files to PNG format using DICOM2PNG, according to the procedure described in Section Image Preparation and Input Representation. The images were subsequently manually annotated under the proposed anatomical-contextual scheme, integrating nine semantic classes corresponding to supporting anatomical structures and the pathological Nodule class. Once the annotation process was completed, the dataset was divided using a 70:30 hold-out strategy, assigning 3500 images to training and 1500 images to internal validation. In addition, an independent external dataset derived from LIDC-IDRI, comprising 700 images, was used for out-of-domain evaluation. During evaluation, a confidence threshold of 0.25 and an IoU threshold of 0.70 were applied uniformly across all architectures and experimental conditions.

The anatomical-contextual scheme comprised nine semantic classes designed to jointly model thoracic anatomy and pulmonary pathology. The supporting anatomical classes included Spinal Cord, Heart A, Heart B, Digestive Region, Left Lung A, Left Lung B, Right Lung A, and Right Lung B, whereas Nodule was defined as a single invariant pathological class independent of its position within the pulmonary parenchyma. This organization allowed the anatomical structures to function as auxiliary spatial references during learning while preserving the pulmonary nodule as the primary pathological target (see Table 4).

The same dataset partition was maintained identically across all architectures and both ablation-study conditions to eliminate variability attributable to differences in training and validation subset composition.

#### 3.1.1. Ablation Study Design

To directly quantify the contribution of the supporting anatomical structures, a controlled ablation study was performed using two training conditions:

- **Nodule-only:** training using exclusively the pathological Nodule class.
- **Nodule + Anatomical Context:** training using the Nodule class together with the eight supporting anatomical classes defined in the proposed framework.

Under both conditions, the same YOLOv8, YOLOv9, YOLOv10, YOLOv11, and YOLOv12 architectures were used while maintaining identical image selection, training–validation partition, input resolution, number of epochs, batch size, optimizer, learning rate, data-augmentation strategies, and evaluation protocol.

Therefore, the presence or absence of the eight supporting anatomical classes constituted the only experimental variable modified between the two training schemes, allowing the effect of anatomical-contextual learning on pulmonary nodule detection to be quantitatively isolated.

Performance under both conditions was evaluated exclusively for the Nodule class using Precision, Recall, F1-score, mAP@50, and mAP@50–95. Consequently, the comparison was not influenced by the performance of the auxiliary anatomical classes and focused directly on determining whether their incorporation during training provided a quantifiable benefit for detection of the primary pathological target.

#### 3.1.2. Common Training Configuration

The five YOLO architectures were trained using a homogeneous hyperparameter configuration, including an image size of 512 × 512 pixels, 100 epochs, a batch size of 8, and optimization using stochastic gradient descent (SGD). Model performance was subsequently evaluated using metrics related to detection Precision, localization capability, and inference time.

The initial learning rate was set to 0.001 and dynamically adjusted according to the scheduling strategy implemented by the training framework. Data-augmentation techniques were also incorporated, including random scaling, horizontal flipping, Mosaic augmentation, and HSV color–space transformations, with the aim of improving learning robustness and reducing overfitting. These parameters correspond to the experimental configuration reported throughout the manuscript.

The hyperparameter configuration shown in Table 5 was kept unchanged across both experimental schemes. Therefore, differences between Nodule-only and Nodule + Anatomical Context can be interpreted without confounding effects from changes in the optimization settings.

#### 3.1.3. Class Imbalance Management

No artificial oversampling, undersampling, class-specific loss weighting, or customized sampling strategy was applied during training. Instead, class distribution was managed primarily during retrospective dataset construction and curation. During selection of the 5000 axial CT slices, an effort was made to maintain adequate representation of the anatomical structures included in the proposed framework while preserving their naturally occurring frequency within the selected thoracic images. Therefore, an exact numerical balance among the nine classes was not artificially imposed. The Nodule and spinal cord classes were among the most frequently represented within the selected slices, whereas the remaining anatomical classes were maintained as homogeneously as reasonably possible according to their actual occurrence in the CT images. No synthetic samples were generated exclusively to equalize class frequencies, and no class-specific loss coefficients were introduced. Consequently, the resulting dataset should be interpreted as having a controlled but non-uniform class distribution that preserves the anatomical and clinical characteristics of the selected CT population.

#### 3.1.4. Loss Function and Optimization Objective

The evaluated YOLOv8m, YOLOv9c, YOLOv10m, YOLOv11m, and YOLOv12m models were implemented using Ultralytics (version 8.3.203) and PyTorch (version 2.5.1). No study-specific loss function or class-dependent weighting scheme was introduced. Instead, the native detection objective was retained to preserve comparability among architectures and between the Nodule-only and Nodule + Anatomical Context experimental conditions.

The overall detection objective was defined as

$$L_{det}=\lambda_{box}L_{CIoU}+{\lambda_{cls}L}_{BCE}+\lambda_{dfl}L_{DFL}$$

where $L_{CIoU}$ represents the bounding-box regression loss based on Complete Intersection over Union (CIoU), $L_{BCE}\;$ corresponds to the Binary Cross-Entropy classification loss, and $L_{DFL}$ and denotes Distribution Focal Loss for distribution-based bounding-box regression. The loss components and weighting coefficients used across the evaluated architectures are summarized in Table 6.

As shown in Table 6, the same weighting coefficients were maintained across YOLOv8m–YOLOv12m and in both experimental conditions, with $\lambda_{box}$ = 7.5, $\lambda_{cls}$ = 0.5 and $\lambda_{dfl}$ = 1.5. Therefore, the introduction of the eight supporting anatomical classes did not involve additional class-specific loss weights or modifications to the optimization objective.

For semantic classification, Binary Cross-Entropy was used to optimize the class predictions. For N supervised predictions and C semantic classes, the classification component can be expressed as

$$L_{BCE}=-\frac{1}{N}\sum_{i=1}^{N}\sum_{c=1}^{C}\left[y_{ic}\log\left({\hat{y}}_{ic}\right)+\left(1-y_{ic}\right)log\left(1-{\hat{y}}_{ic}\right)\right]$$

where ${\hat{y}}_{ic}$ denotes the ground-truth label and the predicted probability for class in prediction. For the anatomical-contextual configuration, C=9, corresponding to eight auxiliary anatomical classes and the invariant pathological Nodule class. In the Nodule-only ablation condition, the same classification formulation was retained with C=1.

Importantly, no additional class-specific loss weight was assigned to the Nodule class or to any supporting anatomical structure; all semantic classes were optimized under the same classification-loss formulation.

Bounding-box localization was optimized using CIoU, which considers overlap between predicted and ground-truth boxes together with center-point distance and aspect-ratio consistency. The corresponding loss can be expressed as

$$L_{CIoU}=1-IoU+\frac{\rho^{2}\left(b,b^{gt}\right)}{c^{2}}+\alpha v$$

where $\rho^{2}\left(b,b^{gt}\right)\;$ denotes the Euclidean distance between the centers of the predicted and ground-truth boxes, c is the diagonal length of the smallest enclosing box containing both boxes, and v and α represent the aspect-ratio consistency term and its adaptive weighting factor, respectively. This component was weighted by $\lambda_{box}=7.5$ during optimization.

Distribution Focal Loss was used to refine bounding-box coordinate regression by representing each box boundary as a discrete probability distribution rather than as a single continuous point estimate. For a target coordinate y located between two adjacent discrete bins $y_{i}\;$ and $y_{i+1}$, the DFL component can be represented as

$$L_{DFL}=-\left[y_{i}-y\right]\log\left(P_{i}\right)+(y-y_{i})log(P_{i+1})$$

where $P_{i}$ and $P_{i+1}$ denote the predicted probabilities associated with the two adjacent bins. Overall, the loss formulation was kept unchanged between the Nodule-only and Nodule + Anatomical Context experiments. Consequently, the anatomical-contextual ablation isolates the effect of incorporating the eight supporting anatomical classes without introducing changes in the loss components or their weighting coefficients.

#### 3.1.5. Statistical Analysis and Reporting

All architectures were evaluated using a fixed hold-out experimental design. The LUNG-PET-CT-DX dataset was partitioned once into 70% training and 30% internal validation subsets, and the identical partition was maintained across YOLOv8m, YOLOv9c, YOLOv10m, YOLOv11m, and YOLOv12m, as well as across the Nodule-only and Nodule + Anatomical Context ablation conditions. Each architecture was trained once under the common experimental configuration described above; therefore, the reported Precision, Recall, F1-score, mAP@50, and mAP@50–95 values represent point estimates obtained from the fixed validation set rather than means across repeated random seeds or cross-validation folds.

Because independent repeated training runs were not performed, standard deviations across seeds were not estimated, and no inferential claim of statistical significance is made when comparing architectures or experimental conditions. Instead, comparisons are reported descriptively using absolute differences in performance metrics under identical experimental conditions. To complement normalized performance measures, absolute confusion-matrix counts were also reported, allowing the magnitude of correct detections and errors to be evaluated together with their relative proportions.

External performance was independently assessed on the LIDC-IDRI subset described in Section 2.5, without retraining, fine-tuning, transfer learning, or domain adaptation. Patient-level confidence intervals were not calculated because the present analysis was based on the predefined slice-level evaluation protocol and patient-clustered resampling was not incorporated into the original experimental design. Future studies should incorporate repeated training runs and patient-level resampling or cross-validation to quantify uncertainty around model performance estimates.

### 3.2. Comparative Analysis of YOLOv8–YOLOv12

This section compares the performance of YOLOv8, YOLOv9, YOLOv10, YOLOv11, and YOLOv12 under the proposed anatomical-contextual framework. The analysis considers pulmonary nodule detection on the internal LUNG-PET-CT-DX validation set and cross-dataset performance on the independent LIDC-IDRI subset.

#### 3.2.1. Comparative Performance on LUNG-PET-CT-DX

The internal LUNG-PET-CT-DX validation set was used to compare the proposed anatomical-contextual framework across different generations of YOLO detectors. Three complementary aspects were examined: training dynamics, quantitative performance for the Nodule class, and qualitative behavior of the multi-organ detections produced by each architecture.

**Figure 11:**
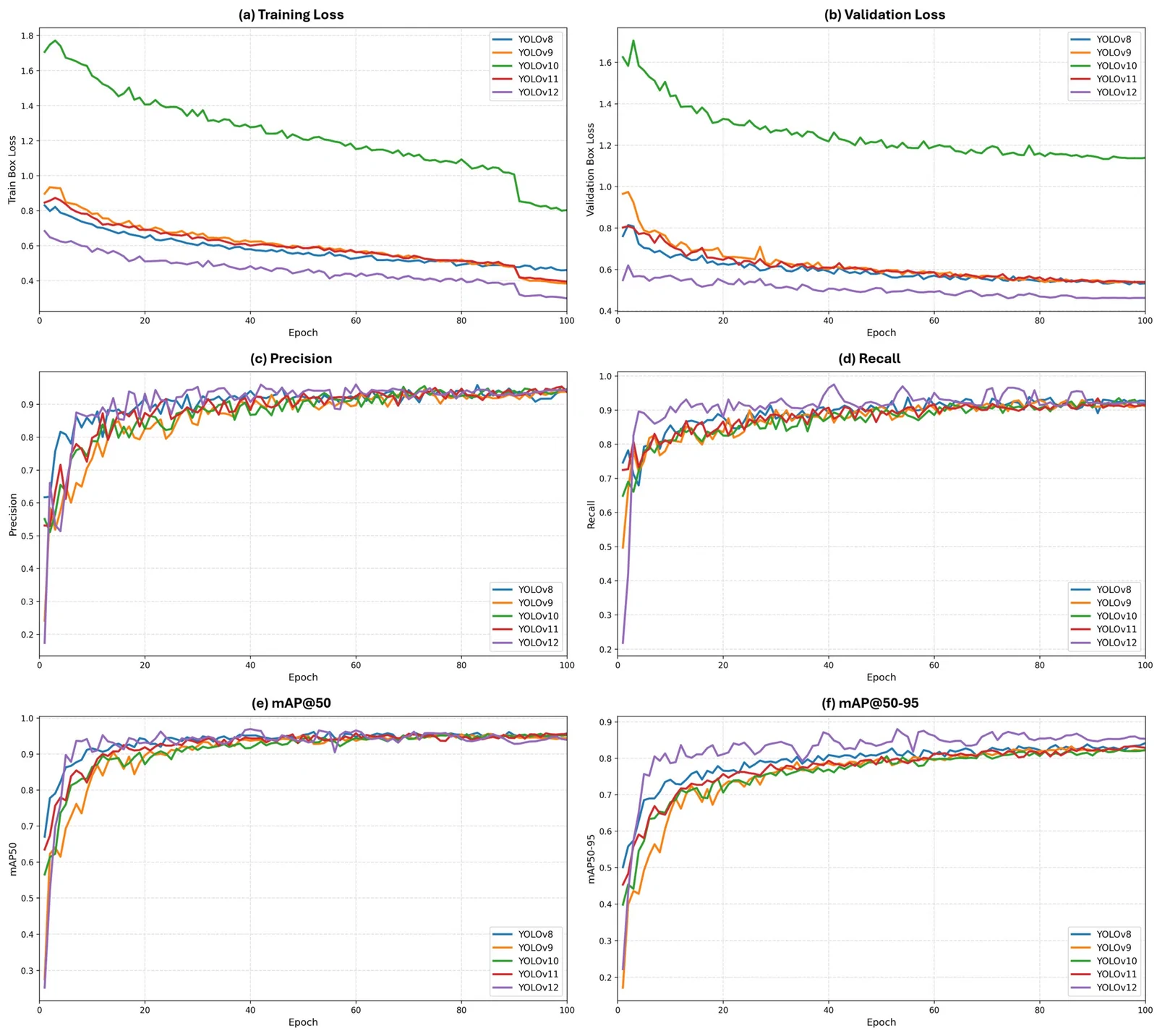
Training loss, validation loss, Precision, Recall, mAP@50, and mAP@50–95 evolution for YOLOv8–YOLOv12 on the internal LUNG-PET-CT-DX validation set under the proposed anatomical-contextual framework.

Figure 11 shows the evolution of the main training metrics for YOLOv8–YOLOv12 over 100 epochs. All five architectures exhibited stable convergence, with no marked divergence during optimization. These results indicate that the anatomical-contextual training scheme was compatible with all evaluated detector generations and did not introduce evident instability during training. This interpretation is consistent with the experimental objective of comparing internal detection capability and subsequent generalization on LIDC-IDRI.

The training and validation loss curves in Figure 11a,b decreased progressively throughout optimization. Their broadly parallel trajectories showed no visual evidence of severe overfitting. YOLOv12 reached the lowest loss values and converged more rapidly during the early epochs; however, this optimization behavior did not translate into superior performance across all final detection metrics.

Among the evaluated architectures, YOLOv12 showed the lowest loss values during most of the optimization process. Training loss decreased from approximately 0.68 to 0.31, while validation loss decreased from approximately 0.60 to 0.46 by epoch 100. YOLOv12 also exceeded 0.90 Precision before epoch 20 and reached mAP@50 values close to 0.93 during the early stages of training. These observations indicate faster early convergence, although they should not be interpreted as evidence of overall superiority across detection metrics.

The Precision, Recall, mAP@50, and mAP@50–95 curves in Figure 11c–f show that all five architectures reached high and relatively stable performance levels. Differences among models were more evident during the early epochs and became smaller as training progressed. By the end of optimization, all architectures achieved competitive performance, indicating that the anatomical-contextual training scheme was effective across multiple YOLO generations rather than being specific to a single architecture.

To complement the interpretation of the training curves, Table 7 summarizes the class-specific performance of each architecture for the Nodule class, defined in this study as the primary pathological target. Unlike Figure 11, which describes the global training dynamics, this table enables direct comparison of pulmonary nodule detection performance using Precision, Recall, F1-score, mAP@50, mAP@50–95, and inference time.

As shown in Table 7, all architectures achieved high performance for the Nodule class. YOLOv10m obtained the highest Precision (0.979) and mAP@50 (0.987), together with the shortest inference time (9.25 ms). In contrast, YOLOv11m achieved the highest Recall (0.971) and F1-score (0.962), indicating the greatest sensitivity among the evaluated models.

These differences may be relevant when selecting a model for a specific application. YOLOv10m offers a favorable combination of Precision, mAP@50, and inference speed, whereas YOLOv11m provides higher sensitivity, which may be advantageous in settings where missed nodules are of particular concern. YOLOv8m also showed strong performance, with a Precision of 0.976 and the highest mAP@50–95 (0.890), indicating comparatively consistent localization across stricter IoU thresholds. YOLOv9c and YOLOv12m remained competitive but did not simultaneously outperform YOLOv10m or YOLOv11m across the main evaluation criteria.

**Figure 12:**
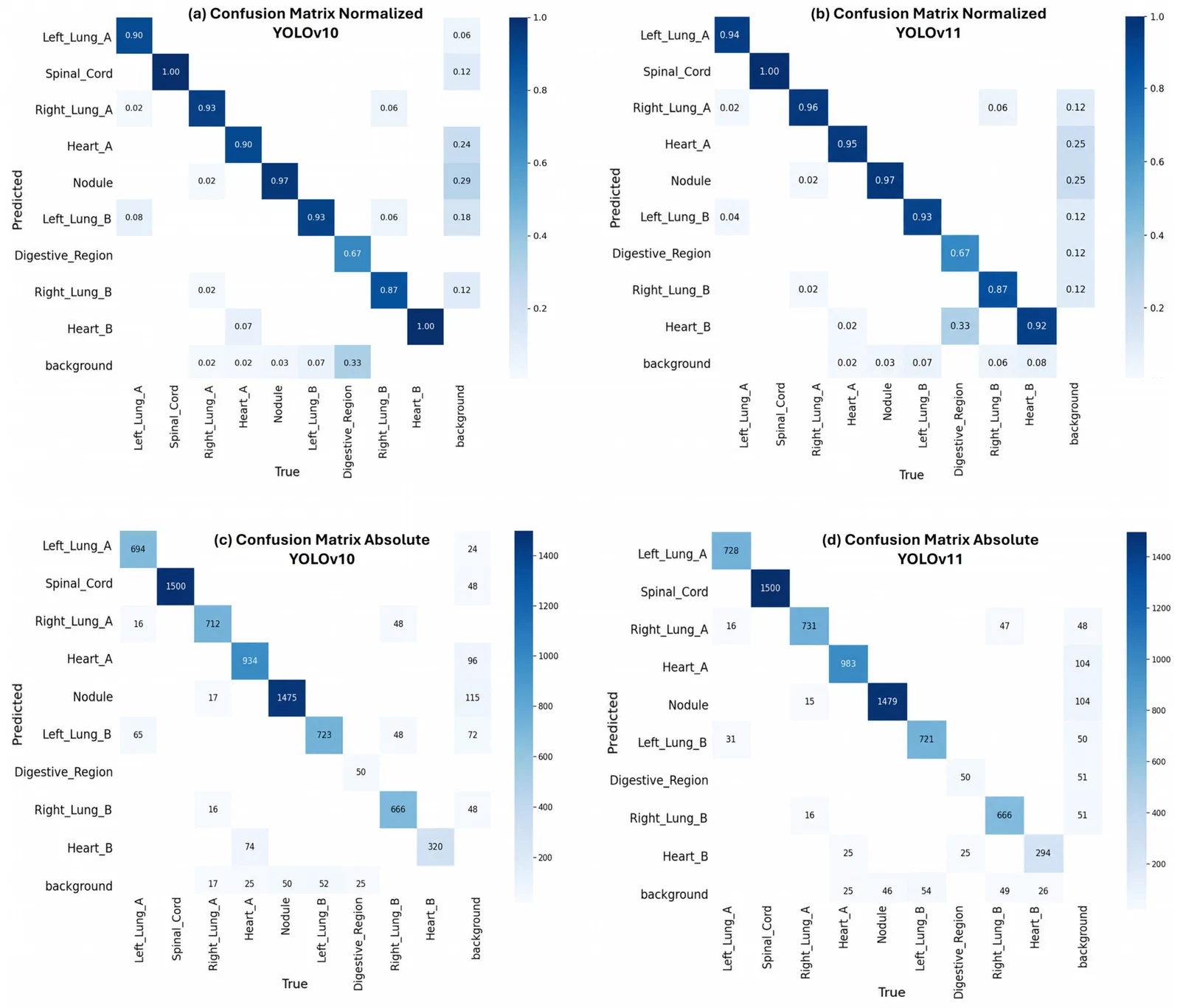
Comparative normalized and absolute confusion matrices for YOLOv10 and YOLOv11 on the internal LUNG-PET-CT-DX validation set. Panels (**a**,**b**) show normalized matrices and panels (**c**,**d**) the corresponding absolute counts. The horizontal axis represents ground-truth classes and the vertical axis predicted classes. Background is used only for error attribution and is not a trained semantic class.

To analyze the class-wise behavior of two complementary high-performing models, Figure 12 presents the normalized and absolute confusion matrices obtained for YOLOv10 and YOLOv11 on the LUNG-PET-CT-DX validation set.

Overall, both matrices show a high concentration of values along the main diagonal, indicating strong class-wise discrimination among the anatomical structures considered and the Nodule class. In YOLOv10, the Nodule class reached a diagonal value of 0.97, whereas Spinal Cord and Heart B reached values of 1.00. Likewise, Left Lung A, Right Lung A, Heart A, Left Lung B, and Right Lung B showed high values, supporting the model’s ability to recognize supporting anatomical structures within the thoracic scene. YOLOv11 shows similar behavior, with Nodule and spinal cord maintaining values of 0.97 and 1.00, respectively, and with relative improvements in some structures such as Left Lung A, Right Lung A, Heart A, and Heart B. The Background category was used exclusively for error attribution and was not included as a tenth trained semantic class. With True classes on the horizontal axis and Predicted classes on the vertical axis, the Background column represents false positives, whereas the Background row represents false negatives.

Although overall performance was high, the digestive region remained the most challenging anatomical class, with diagonal values of approximately 0.67 and recurrent confusion with Heart B. This pattern is consistent with the anatomical complexity of the inferior thoracoabdominal region, where morphological variability and spatial proximity among adjacent structures may increase visual ambiguity. These errors were largely confined to neighboring anatomical classes and did not substantially affect recognition of the Nodule class.

**Figure 13:**
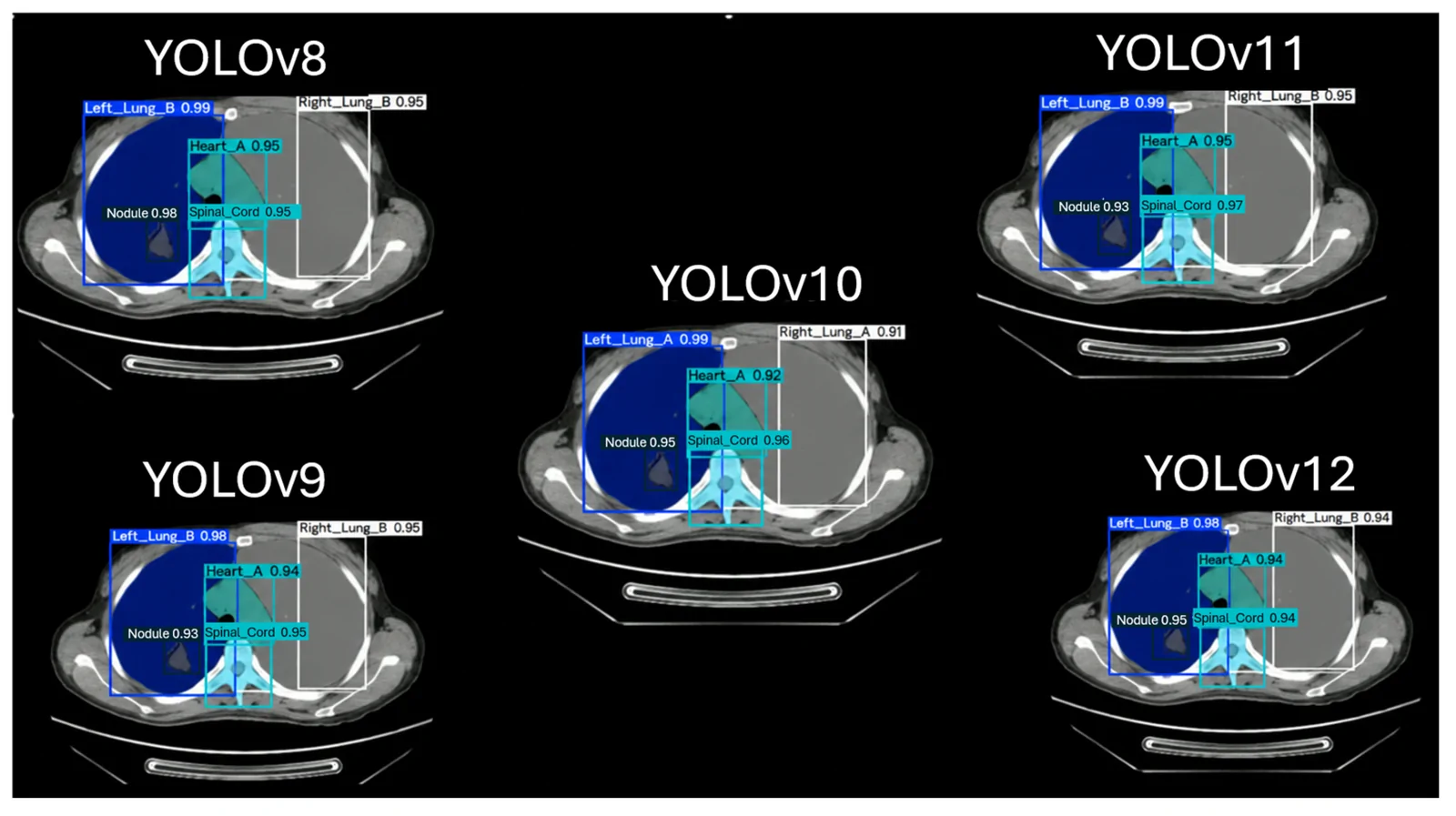
Qualitative multi-organ detection results obtained with YOLOv8–YOLOv12 on representative CT slices from the internal LUNG-PET-CT-DX validation set. The figure illustrates simultaneous detection of thoracic support structures and pulmonary nodule localization under the proposed anatomical-contextual framework.

To qualitatively illustrate this behavior, Figure 13 presents a visual comparison of the inferences obtained by YOLOv8, YOLOv9, YOLOv10, YOLOv11, and YOLOv12 on representative slices from the LUNG-PET-CT-DX dataset.

Figure 13 shows that all five architectures simultaneously detected the Nodule class and supporting anatomical structures, including the pulmonary regions, heart, and spinal cord. These qualitative examples illustrate how the proposed framework places nodule localization within a broader anatomical representation rather than treating the lesion as an isolated object.

Taken together, Table 7 and Figure 11, Figure 12 and Figure 13 show that no architecture was consistently superior across all criteria. YOLOv10m showed the strongest combination of Precision, mAP@50, and computational efficiency; YOLOv11m achieved the highest Recall and F1-score; and YOLOv8m provided the highest mAP@50–95. These findings support model selection based on the required balance between sensitivity, Precision, localization consistency, and computational efficiency rather than on detector generation alone.

Finally, the results support the hypothesis that the explicit incorporation of multi-organ anatomical context favors the learning of spatial relationships relevant to pulmonary nodule detection. The proposed framework enabled robust performance across different generations of detectors, maintaining stable detection of the Nodule class and a coherent anatomical representation of the supporting thoracic structures. This evidence justifies the subsequent external evaluation on LIDC-IDRI, aimed at determining whether the representations learned on LUNG-PET-CT-DX preserve stability under a domain shift.

#### 3.2.2. Ablation Study: Nodule-Only vs. Nodule + Anatomical Context

To directly quantify the contribution of anatomical context to pulmonary nodule detection, an ablation study was conducted comparing two training conditions: Nodule-only, in which the models were trained exclusively using the Nodule class, and Nodule + Anatomical Context, corresponding to the proposed framework incorporating eight auxiliary anatomical classes. According to the protocol described in Section 3.1.1, both conditions used the same architectures, dataset partition, input resolution, hyperparameters, optimization strategy, and evaluation criteria, such that the presence or absence of anatomical context constituted the only experimental variable modified.

Table 8 presents the results obtained on the internal LUNG-PET-CT-DX validation set. Across all five evaluated architectures, the incorporation of anatomical context produced consistent improvements in Precision, Recall, F1-score, mAP@50, and mAP@50–95 compared with their corresponding Nodule-only configurations. This cross-architecture pattern indicates that the benefit of anatomical context was not restricted to a specific YOLO generation.

The largest gains in Precision and mAP@50 were observed for YOLOv10m (+0.114 and +0.100, respectively). YOLOv11m showed the greatest improvements in Recall (+0.165) and F1-score (+0.116), while YOLOv12m also showed a substantial increase in Recall (+0.157). These results indicate that anatomical context improved both the reliability of positive detections and the proportion of nodules successfully identified.

The largest improvements were observed in mAP@50–95, reaching +0.219 for YOLOv9c, +0.214 for YOLOv8m, and +0.211 for YOLOv11m. Because mAP@50–95 evaluates performance across progressively stricter IoU thresholds, these gains suggest improved localization consistency when anatomical context was incorporated during training.

Across the five architectures, the anatomical-contextual scheme produced mean absolute improvements of +0.078 in Precision, +0.122 in Recall, +0.101 in F1-score, +0.079 in mAP@50, and +0.190 in mAP@50–95. The consistency of these gains across different architectures provides direct experimental evidence that supporting anatomical structures provide complementary information for learning the Nodule class.

Overall, the ablation study supports the central hypothesis of this work: incorporating multi-organ anatomical context consistently improved pulmonary nodule detection compared with Nodule-only training. The largest average gains were observed in Recall and mAP@50–95, suggesting benefits in both nodule recovery and localization consistency.

#### 3.2.3. Cross-Dataset Generalization on LIDC-IDRI

To assess cross-dataset generalization, YOLOv8–YOLOv12 models trained exclusively on LUNG-PET-CT-DX were evaluated directly on an independent LIDC-IDRI subset comprising 700 axial CT slices from 70 patients. No retraining, fine-tuning, transfer learning, or domain adaptation was performed. This evaluation tested whether representations learned from LUNG-PET-CT-DX remained useful under an external CT distribution characterized by differences in acquisition and patient anatomy.

**Figure 14:**
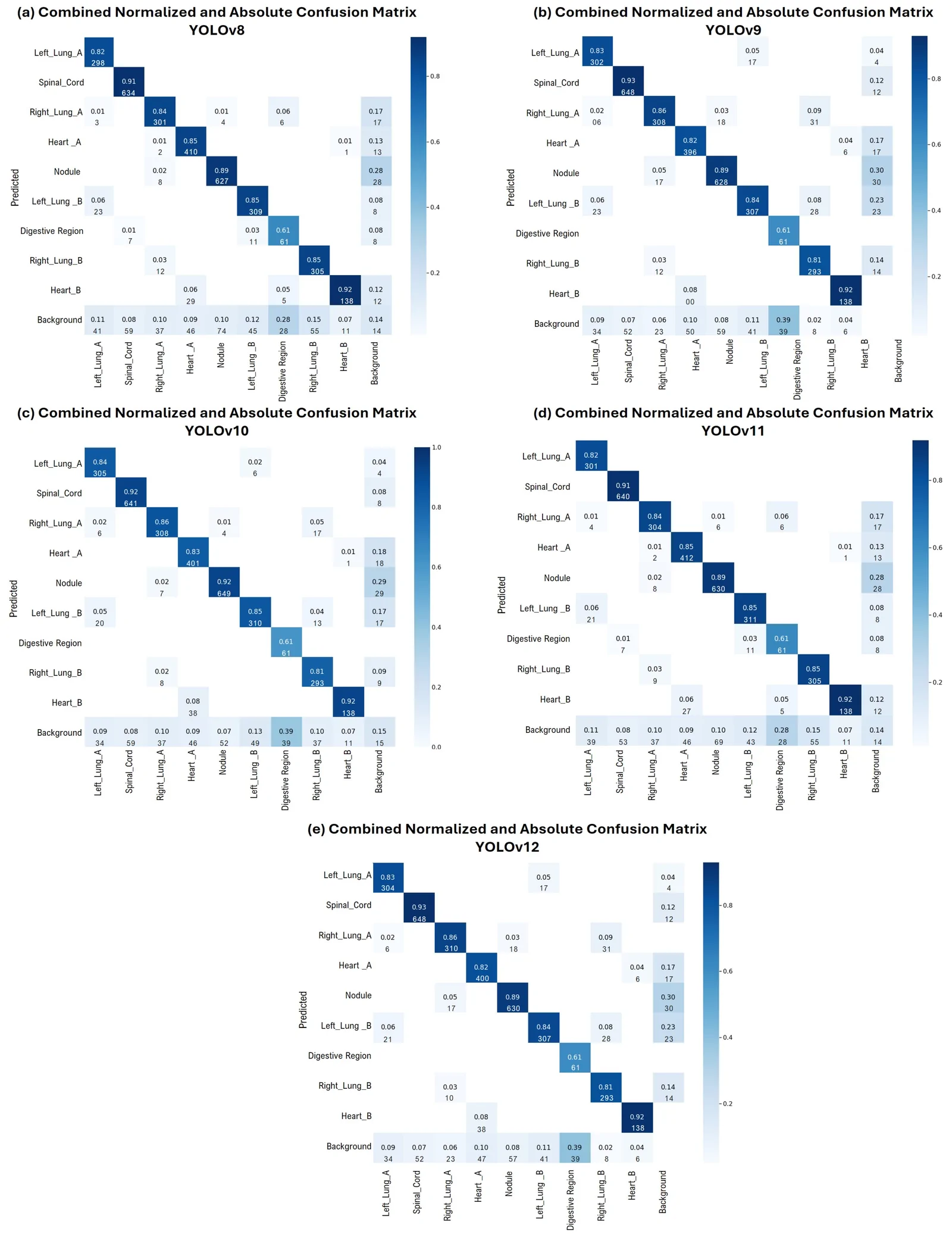
Combined normalized and absolute confusion matrices for YOLOv8–YOLOv12 during external evaluation on the LIDC-IDRI subset. Panels (**a**–**e**) correspond to YOLOv8, YOLOv9, YOLOv10, YOLOv11, and YOLOv12, respectively; each populated cell shows the normalized proportion above and the absolute count below. The eight anatomical classes represent auxiliary contextual references, whereas Nodule is the primary pathological target. Background is included only for error attribution and is not a trained class.

As shown in Figure 14, the five architectures preserved a predominant concentration along the main diagonal of the combined normalized and absolute confusion matrices. Each populated cell reports both the normalized class-wise proportion and the corresponding absolute number of instances, allowing the relative recognition pattern to be interpreted together with its underlying count. These diagonal values correspond to the fixed operating point used to construct the confusion matrices and should therefore not be interpreted as direct numerical equivalents of the aggregate Precision, Recall, F1-score, or mAP values reported in Table 9.

The Nodule class, corresponding to the primary pathological target, maintained stable diagonal recognition across the evaluated architectures. YOLOv10 showed the highest diagonal value for this class, reaching 0.92 with 649 correctly assigned instances. YOLOv8, YOLOv9, YOLOv11, and YOLOv12 each presented a diagonal value of approximately 0.89, corresponding to 627, 628, 630, and 630 instances, respectively. This pattern is broadly consistent with the nodule-specific results reported in Table 9, while small differences between the matrix diagonal and the aggregate Recall values reflect the different evaluation summaries used by the confusion-matrix operating point and the overall detection metrics.

Within the confusion-matrix analysis of the supporting anatomical structures, Heart B showed particularly stable recognition, with a diagonal value of 0.92 and 138 instances across all five architectures, while Spinal Cord remained between 0.91 and 0.93. In contrast, Digestive Region consistently presented the lowest diagonal value (0.61; 61 instances), confirming the greater difficulty associated with the inferior thoracoabdominal region. Basal pulmonary regions also exhibited moderate variability among architectures, consistent with their morphological heterogeneity and spatial proximity to adjacent structures. As in the internal confusion matrices, True classes are shown on the horizontal axis and Predicted classes on the vertical axis; therefore, the Background column represents false positives, whereas the Background row represents false negatives.

Because the eight anatomical classes functioned exclusively as auxiliary contextual references, differences in their class-specific recognition were not interpreted as independent clinical failures. In particular, the lower recognition observed for the Digestive Region reflects greater variability in an auxiliary contextual class and does not directly indicate reduced pulmonary nodule detection performance. The principal external evaluation endpoint remained the Nodule class, whose Precision, Recall, F1-score, mAP@50, and mAP@50–95 values are reported separately in Table 9.

To complement the confusion-matrix interpretation, Table 9 presents the Nodule-specific performance obtained during external evaluation on LIDC-IDRI. Unlike Figure 14, which focuses on recognition and confusion patterns among classes, the table summarizes the quantitative behavior of the primary pathological target using Precision, Recall, F1-score, mAP@50, mAP@50–95, and inference time. All results correspond to models trained under the Nodule + Anatomical Context scheme and evaluated on LIDC-IDRI without retraining, fine-tuning, or domain adaptation.

The results in Table 9 show that all five architectures maintained competitive performance for the Nodule class when evaluated on an external tomographic distribution not used during training. YOLOv10m achieved the highest Precision (0.924) and mAP@50 (0.956), together with the lowest inference time (9.25 ms). Therefore, YOLOv10m maintained a favorable combination of Precision, mAP@50, and computational efficiency under domain shift.

In contrast, YOLOv8m achieved the highest Recall (0.930) and mAP@50–95 (0.845), indicating stronger sensitivity and comparatively consistent localization across stricter IoU thresholds. YOLOv8m and YOLOv10m achieved the same F1-score (0.922) but through different performance profiles: YOLOv8m favored sensitivity, whereas YOLOv10m favored Precision.

This pattern may be related to the localization characteristics of YOLOv8m, whose anchor-free decoupled detection head and multiscale FPN/PAN feature aggregation may support stable bounding-box localization across different object scales. Under the domain shift introduced by LIDC-IDRI, these features may have contributed to maintaining localization performance as the IoU threshold increased. However, because the present study was not designed to isolate individual architectural components, this interpretation should be regarded as a plausible explanation rather than evidence of a causal advantage of YOLOv8m.

The remaining architectures showed intermediate performance. YOLOv9c achieved a Precision of 0.884, Recall of 0.900, F1-score of 0.892, mAP@50 of 0.934, and mAP@50–95 of 0.838, although it exhibited the highest inference time (16.13 ms). YOLOv11m and YOLOv12m both achieved a Recall of 0.890, with F1-scores of 0.882 and 0.883, respectively. Although YOLOv12m achieved an mAP@50 of 0.937, it showed the lowest mAP@50–95 value (0.792), indicating lower relative localization consistency under stricter overlap criteria.

Overall, no architecture was superior across all external evaluation criteria. YOLOv10m achieved the highest Precision, mAP@50, and computational efficiency, whereas YOLOv8m achieved the highest Recall and mAP@50–95 while sharing the highest F1-score with YOLOv10m. These results provide evidence of cross-dataset generalization within the evaluated LIDC-IDRI subset but should not be interpreted as definitive clinical validation.

**Figure 15:**
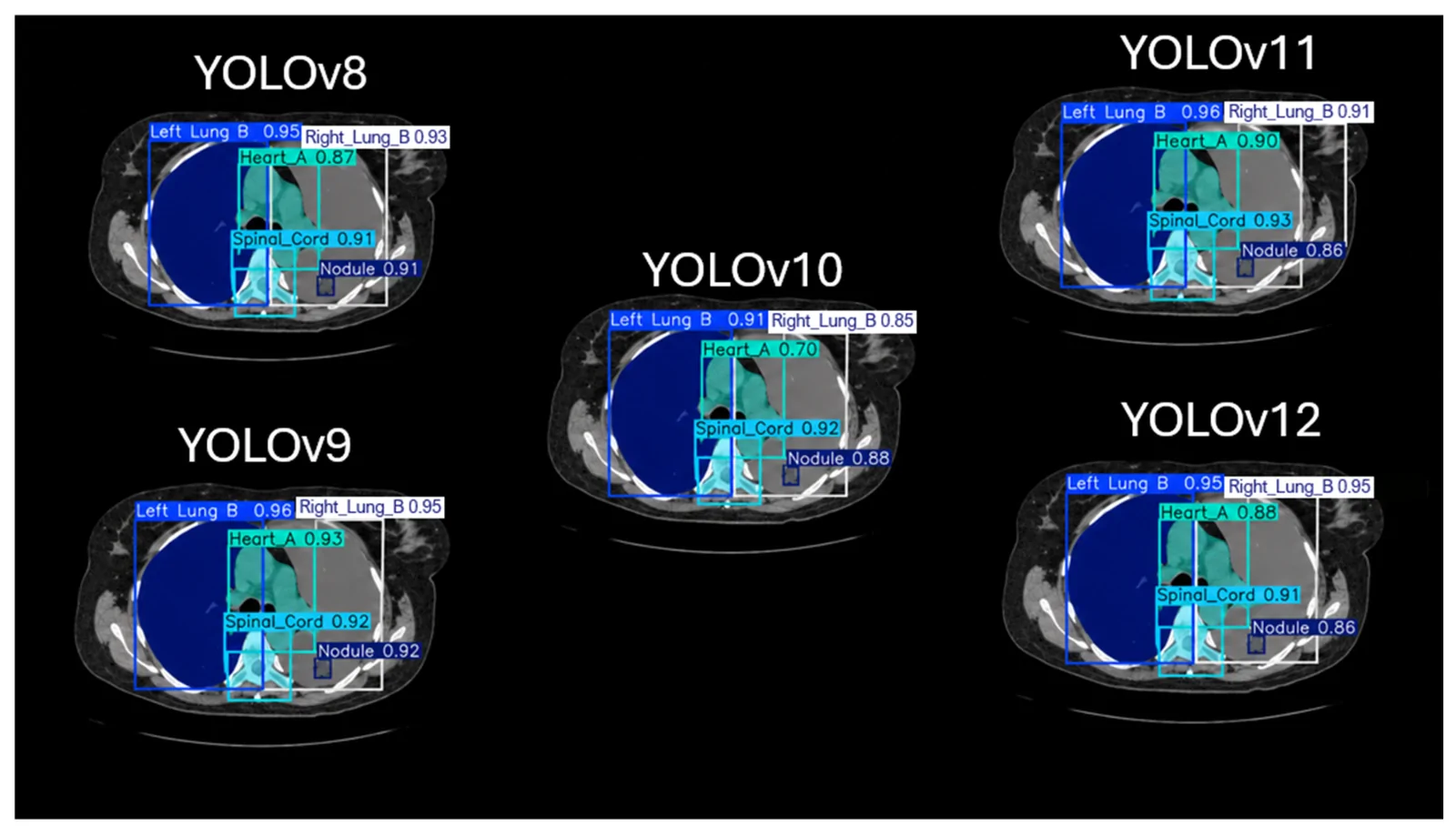
Qualitative multi-organ detection results obtained with YOLOv8–YOLOv12 on a representative CT slice from the external LIDC-IDRI dataset. The figure illustrates pulmonary nodule localization and simultaneous detection of anatomical support structures under cross-dataset evaluation without fine-tuning or retraining.

Figure 15 provides qualitative examples of inference on LIDC-IDRI. All five architectures localized the pulmonary nodule while also predicting the supporting anatomical structures, illustrating that anatomical-contextual patterns learned from LUNG-PET-CT-DX were retained when applied to an external dataset. These examples should be interpreted as complementary visual evidence; cross-dataset generalization is supported primarily by the quantitative results reported in Table 9.

## 4. Discussion and Conclusions

### 4.1. Principal Findings

The findings of this study show that the proposed multi-organ anatomical-contextual framework frames pulmonary nodule detection as a spatial interpretation task within the thoracic cavity rather than as isolated local lesion recognition. This distinction is relevant because pulmonary nodules coexist with anatomically complex structures on CT, particularly in basal, mediastinal, and diaphragmatic regions, where visual similarities may increase detection ambiguity.

The principal finding derives from the controlled ablation study. Under identical experimental conditions, incorporating the eight supporting anatomical classes consistently improved Precision, Recall, F1-score, mAP@50, and mAP@50–95 across all five architectures compared with their corresponding Nodule-only configurations. On average, the Nodule + Anatomical Context scheme yielded absolute gains of +0.078 in Precision, +0.122 in Recall, +0.101 in F1-score, +0.079 in mAP@50, and +0.190 in mAP@50–95. The consistency of these improvements across multiple YOLO generations provides direct experimental evidence that anatomical context contributes complementary information to pulmonary nodule detection and localization.

The results presented in Section 3 also show that all five architectures achieved competitive performance during both internal validation on LUNG-PET-CT-DX and external evaluation on LIDC-IDRI. However, no single architecture was superior across all metrics. During internal validation, YOLOv10m achieved the highest Precision and mAP@50 together with the shortest inference time, YOLOv11m achieved the highest Recall and F1-score, and YOLOv8m achieved the highest mAP@50–95, indicating comparatively consistent localization under stricter IoU thresholds.

A similar distribution of strengths was observed during external evaluation. YOLOv10m retained the highest Precision (0.924) and mAP@50 (0.956), together with the shortest inference time, whereas YOLOv8m achieved the highest Recall (0.930) and mAP@50–95 (0.845). Both architectures achieved an F1-score of 0.922. These findings indicate that architecture selection should reflect application-specific priorities, such as sensitivity, Precision, localization consistency, and computational efficiency, rather than detector generation alone.

### 4.2. Impact of Anatomical-Contextual Learning

The main contribution of this study extends beyond the comparison of different YOLO generations by showing that anatomical context can provide complementary spatial information for pulmonary nodule detection. Incorporating structures such as the spinal cord, Heart A, Heart B, digestive region, and the differentiated pulmonary regions provides the detector with anatomical references that complement the local characteristics of the lesion.

The ablation study directly supports this interpretation. The Nodule + Anatomical Context scheme consistently improved all five evaluated metrics compared with Nodule-only training across every architecture. The largest average gains were observed in Recall and mAP@50–95, suggesting that anatomical context contributes both to identifying a greater proportion of nodules and to maintaining more consistent localization. Because this pattern was observed across multiple YOLO generations, the benefit was not limited to a specific detector architecture.

From a methodological perspective, this effect may be interpreted as a form of anatomical regularization. Rather than relying exclusively on local texture, contrast, borders, or morphology, the detector learns the lesion in relation to surrounding thoracic structures. The improvement in mAP@50–95 suggests greater localization consistency under stricter IoU criteria, whereas the increase in Recall indicates improved recovery of nodules that were missed when training was restricted to the pathological class alone.

The confusion matrices provide complementary evidence for this interpretation. Nodule recognition remained stable, whereas most classification errors were concentrated in anatomically variable regions, particularly the digestive region and basal thoracic areas. These findings suggest that incorporating supporting anatomical structures does not compromise detection of the primary pathological target and instead promotes a more structured representation of the thoracic scene.

### 4.3. Comparative Behavior of YOLOv8–YOLOv12

Comparison of YOLOv8–YOLOv12 showed that newer detector generations did not produce a monotonic improvement in pulmonary nodule detection performance. Under the same Nodule + Anatomical Context scheme and identical experimental conditions, each architecture exhibited distinct strengths, indicating that performance depends on the interaction between architectural design and the evaluation metric rather than on model recency alone.

During internal validation on LUNG-PET-CT-DX, YOLOv10m achieved the highest Precision (0.979), mAP@50 (0.987), and computational efficiency, with an inference time of 9.25 ms. YOLOv11m achieved the highest Recall (0.971) and F1-score (0.962), whereas YOLOv8m achieved the highest mAP@50–95 (0.890). No architecture therefore provided the best sensitivity, Precision, localization consistency, and computational efficiency simultaneously.

The same pattern was observed during external evaluation on LIDC-IDRI. YOLOv10m retained the highest Precision (0.924), mAP@50 (0.956), and shortest inference time (9.25 ms), whereas YOLOv8m achieved the highest Recall (0.930) and mAP@50–95 (0.845). Both models achieved an F1-score of 0.922 but through different performance profiles. These findings support a multicriteria approach to architecture selection in CAD systems, in which sensitivity, Precision, localization consistency, and computational requirements are considered according to the intended application.

### 4.4. Discussion of Cross-Dataset Generalization on LIDC-IDRI 

External evaluation on LIDC-IDRI provided evidence of cross-dataset generalization under the evaluated domain shift. The models were trained exclusively on LUNG-PET-CT-DX and subsequently evaluated on an independent subset comprising 700 axial slices from 70 patients, without retraining, fine-tuning, transfer learning, or domain adaptation.

Despite the change in data distribution, all five architectures maintained competitive performance for the Nodule class. YOLOv10m achieved the highest Precision (0.924) and mAP@50 (0.956), whereas YOLOv8m achieved the highest Recall (0.930) and mAP@50–95 (0.845); both models achieved an F1-score of 0.922. These results indicate that representations learned under the Nodule + Anatomical Context scheme retained useful information for pulmonary nodule detection when applied to the evaluated external dataset.

Nevertheless, these findings should be interpreted as evidence of cross-dataset generalization within the evaluated LIDC-IDRI subset rather than as definitive clinical validation. The analysis was performed on selected 2D slices rather than complete LIDC-IDRI volumes or a prospective multicenter cohort. For the Nodule class, the external reference was derived from the original multi-reader LIDC-IDRI radiological annotations. The eight supporting anatomical classes were evaluated against study-specific expert-reviewed annotations generated exclusively for the external test subset. Their performance therefore provides complementary information regarding preservation of the learned anatomical representation, while pulmonary nodule detection remains the primary pathological endpoint of the study.

### 4.5. Limitations and Future Work

This study presents several limitations that should be considered when interpreting the results. First, although external evaluation was performed on an independent subset of LIDC-IDRI, the analysis was limited to 700 axial CT slices from 70 patients rather than complete volumetric studies or a prospective multicenter cohort. Therefore, the external results should be interpreted as evidence of cross-dataset generalization within the evaluated subset rather than as definitive clinical validation. Future studies should extend the evaluation to larger multicenter populations and complete CT examinations.

Second, the proposed framework operates on individual 2D axial slices and therefore does not explicitly model inter-slice continuity, volumetric shape, or multiplanar information. This limitation is particularly relevant for pulmonary vessels oriented along the craniocaudal axis, because their transverse appearance in an isolated axial slice may resemble a small pulmonary nodule and potentially contribute to false-positive detections. The anatomical-contextual representation may reduce some of this ambiguity by providing structured information about the surrounding thoracic scene; however, it cannot replace the longitudinal information available from consecutive slices. The 2D design was selected to enable a homogeneous comparison across YOLOv8–YOLOv12 architectures while preserving computational efficiency and compatibility with fast slice-level inference. Future work should therefore investigate 2.5D and 3D strategies capable of integrating adjacent slices, volumetric continuity, and multiplanar context.

Third, because the present evaluation was performed on selected 2D slices rather than complete CT volumes containing both positive and negative slices, scan-level free-response receiver operating characteristic (FROC) curves and false positives per scan or per volume were not estimated. These metrics require evaluation over complete examinations and are particularly important for assessing the clinical burden of false-positive detections in CAD systems. Future studies should therefore include full-volume inference and report FROC performance and false-positive rates per CT examination.

Performance was not stratified according to clinically relevant nodule characteristics such as size, density, or morphology. Although the original polygonal annotations preserved the lesion contour during labeling, the evaluated YOLO models operated on bounding-box representations derived from those polygons. Therefore, no volumetric nodule size in mm^3^ was calculated, and the annotation protocol did not distinguish radiological subtypes such as solid, subsolid, ground-glass, calcified, or spiculated nodules. Future studies should incorporate clinically defined size and morphology strata to determine whether the anatomical-contextual benefit is preserved across lesions of different radiological complexity.

Finally, although the controlled ablation study demonstrated a consistent benefit of anatomical-contextual learning over Nodule-only training, the clinical utility of this representation still requires reader-based evaluation. Future studies should assess whether the proposed framework can reduce interpretation time, improve diagnostic confidence, decrease missed nodules, and maintain robust performance under real-world human-in-the-loop conditions.

Additionally, each architecture was evaluated using a single training run and a fixed hold-out partition. Consequently, variability across random seeds and patient-level confidence intervals were not estimated, and the reported inter-model and ablation differences should be interpreted as descriptive comparisons under controlled experimental conditions rather than formal statistical significance tests. Future studies should incorporate repeated runs and patient-level resampling strategies to quantify uncertainty and reproducibility.

### 4.6. Conclusions

This study presented a multi-organ anatomical-contextual framework for pulmonary nodule detection in axial CT images using YOLOv8–YOLOv12 architectures. By incorporating supporting thoracic structures as spatial references while preserving the pulmonary nodule as a single invariant pathological class, the proposed approach enables lesion detection within a structured anatomical representation rather than as an isolated local target.

The controlled ablation study showed that Nodule + Anatomical Context consistently outperformed Nodule-only training across all five architectures, with mean absolute improvements of +0.078 in Precision, +0.122 in Recall, +0.101 in F1-score, +0.079 in mAP@50, and +0.190 in mAP@50–95. These results provide direct experimental evidence that supporting anatomical structures contribute complementary information to pulmonary nodule detection.

During internal validation, YOLOv10m achieved the highest Precision (0.979), mAP@50 (0.987), and computational efficiency, with an inference time of 9.25 ms; YOLOv11m achieved the highest Recall (0.971) and F1-score (0.962); and YOLOv8m achieved the highest mAP@50–95 (0.890). During external evaluation on LIDC-IDRI, YOLOv10m maintained the highest Precision (0.924) and mAP@50 (0.956), whereas YOLOv8m achieved the highest Recall (0.930) and mAP@50–95 (0.845); both models achieved an F1-score of 0.922.

Overall, the findings support anatomical-contextual learning as a promising strategy for improving pulmonary nodule detection while maintaining competitive performance across datasets. However, the external results should be interpreted as evidence of generalization within the evaluated LIDC-IDRI subset rather than as definitive clinical validation. Future studies should extend evaluation to complete CT volumes and larger multicenter cohorts, incorporate volumetric information, assess performance across clinically relevant nodule characteristics, and evaluate the clinical utility of the framework through reader-based studies.

## Figures and Tables

**Table 1:** Comparative overview of recent deep learning-based approaches for pulmonary nodule detection and the proposed anatomical-contextual YOLOv8–YOLOv12 framework. For the proposed framework, the metrics shown correspond to YOLOv10m on the internal LUNG-PET-CT-DX validation set; external results on LIDC-IDRI are reported separately in Section 3.2.3.

Author	Dataset	Imaging Technique	Approach	Model	Reported Metrics
Zeng et al., 2025 [33]	LUNG-PET-CT-DX; LUNA16	CT; PET-CT	Pulmonary nodule detection	YOLO-ED (YOLOv8-based)	Precision: 0.926 Recall: 0.903 mAP@50: 0.947
Lan et al., 2026 [22]	LungCT-Roboflow Dataset; LUNA16	CT	Pulmonary nodule detection	WTAM-YOLO (YOLOv11-based)	**Roboflow:** Precision: 0.944; Recall: 0.872; mAP@50: 0.950. **LUNA16:** mAP@50: 0.931; Recall: 0.915
Li et al., 2026 [15]	LIDC-IDRI; LUNA16	CT	Pulmonary nodule detection	HARM-YOLO (YOLOv10-based)	**LIDC-IDRI:** Precision: 0.897; Recall: 0.874; mAP@50: 0.908. **LUNA16:** Precision: 0.912; Recall: 0.889; mAP@50: 0.925
Song et al., 2025 [34]	LUNA16; Node21 Dataset; LungCT Dataset	CT	Small pulmonary nodule detection	Improved YOLOv11 (Spatial-SE and EAPIoU loss)	**LUNA16:** Precision: 0.781; Recall: 0.773; mAP@50: 0.755. **Node21:** accuracy/detection accuracy: 0.891. **LungCT:** mAP@50: 0.924
Luo et al., 2025 [35]	LUNA25	CT	Pulmonary nodule detection	YOLOv11 + SACL	Precision: 0.764; Recall: 0.634; mAP@50: 0.696
Shuvo et al., 2026 [36]	LUNA16; Author-Curated CT Dataset	CT	Early diagnosis of lung cancer	3D-Res-U-Net; YOLOv5; ViT	Precision: 0.962 (ViT); mAP@50: 0.76 (YOLOv5)
Samaras et al., 2026 [37]	LIDC-IDRI; Custom PET-CT Dataset	CT; PET-CT	Solitary pulmonary nodule classification	Explainable YOLOv8	Precision: 0.962; Recall: 0.87; mAP@50: 0.908
Proposed 2026	LUNG-PET-CT-DX; LIDC-IDRI	CT	Anatomical-contextual pulmonary nodule detection with multi-organ spatial references and cross-dataset evaluation	YOLOv8m; YOLOv9c; YOLOv10m; YOLOv11m; YOLOv12m	Internal validation, YOLOv10m: Precision: 0.979; Recall: 0.935; mAP@50: 0.987. External LIDC-IDRI performance is reported separately in Section 3.2.3.

**Table 2:** Description of the LUNG-PET-CT-DX dataset used for training and internal validation.

Category	Feature	Description
**Dataset** **information**	Dataset name	LUNG-PET-CT-DX [38]
	Independent external evaluation and cross-dataset generalization	Training and internal validation
	Imaging modality	Thoracic CT
	Source image format	DICOM
	Experimental image format	PNG converted from DICOM using DICOM2PNG (version 2023.12.31)
	Number of images in the original dataset	251,135
**Composition of the training-validation dataset**	Number of patients	320
	Total axial CT slices for experimentation	5000
	Slice selection criterion	Controlled representation of anatomical and pathological classes without enforcing exact numerical balance
	Training set	3500 axial slices
	Internal validation set	1500 axial slices
	Validation strategy	Hold-out validation
	Split ratio	70% training/30% validation
**Image characteristics**	Resolution	512 × 512 pixels
	Pixel spacing	Approximately 1 mm × 1 mm
	Slice thickness	2 mm
	Slice interval	0.625–5 mm
	Mediastinal window	Width: 350 HU; Level: 40 HU
	Pulmonary window	Width: 1400 HU; Level: −700 HU
**Anatomical** **annotation** **strategy**	Annotation type	Manual anatomical-contextual labeling
	Annotation format	Polygon-based anatomical annotations
	Support structures	Spinal cord, heart and digestive system
	Pulmonary structures	Left and right lungs
	Axial pulmonary subclasses	Left_Lung_A/B and Right_Lung_A/B
	Pulmonary nodule representation	Single invariant pathological class
**Framework** **configuration**	Total classes	9
	Annotation platform	Manual annotation using LabelMe
	Clinical reference	Thoracic radiologist annotations
	Methodological contribution	Anatomically conditioned multi-organ representation learning

**Table 3:** Description of the LIDC-IDRI dataset used for independent external evaluation.

Category	Feature	Description
**Dataset** **information**	Dataset name	LIDC-IDRI [39]
	Experimental role	Independent external evaluation and cross-dataset generalization
	Imaging modality	Thoracic CT
	Original dataset size	1018 CT studies
**Composition of the test dataset**	Selected patients	70
	Selected axial CT slices	700
	Selection strategy	10 representative axial slices per patient
	Total classes	9: one pathological target and eight auxiliary anatomical-context classes
**Annotation** **reference**	Nodule reference	Original LIDC-IDRI XML annotations generated through the two-phase reading process involving four experienced thoracic radiologists
	Anatomical reference	Study-specific expert-reviewed annotations for the eight auxiliary anatomical-context classes
	Quality control	Two-stage quality control: complete secondary biomedical review followed by final radiological supervision and validation
**Image characteristics**	Resolution	512 × 512 pixels
	Pixel spacing	Approximately 1 mm × 1 mm
	Slice thickness	2 mm
	Slice interval	0.625–5 mm
**Experimental** **use**	Training participation	Not used for training, internal validation, fine-tuning, or domain adaptation
	Evaluation protocol	External inference without retraining
	Evaluation objective	Assessment of cross-dataset generalization under an external CT distribution

**Table 4:** Nine semantic classes used in the anatomical-contextual framework, including eight supporting thoracic structures and one invariant pathological target.

ID	Class	Experimental Function
**1**	Spinal Cord	Posterior anatomical reference
**2**	Heart A	Upper/mid cardiac anatomical representation
**3**	Heart B	Lower cardiac anatomical representation
**4**	Digestive Region	Inferior anatomical reference
**5**	Left Lung A	Upper left lung region
**6**	Left Lung B	Lower left lung region
**7**	Right Lung A	Upper right lung region
**8**	Right Lung B	Lower right lung region
**9**	Nodule	Invariant pathological target

**Table 5:** Common hyperparameter configuration used for training the YOLO models under the Nodule-only and Nodule + Anatomical Context schemes.

YOLO Model	Epochs	Patience	Pretrained	Input Size	lr0	lrf	Batch	Dropout
YOLOv8m	100	100	true	512 × 512	0.001	0.01	8	0.0
YOLOv9c	100	100	true	512 × 512	0.001	0.01	8	0.0
YOLOv10m	100	100	true	512 × 512	0.001	0.01	8	0.0
YOLOv11m	100	100	true	512 × 512	0.001	0.01	8	0.0
YOLOv12m	100	100	true	512 × 512	0.001	0.01	8	0.0

**Note:** The same hyperparameters and experimental conditions were used under the Nodule-only and Nodule + Anatomical Context schemes. The only difference between the two conditions was the presence or absence of the eight supporting anatomical classes.

**Table 6:** Loss components and weighting coefficients used during training of YOLOv8m–YOLOv12m. The same loss formulation and coefficients were applied across all evaluated architectures and in both Nodule-only and Nodule + Anatomical Context experimental conditions.

YOLO Model	Framework	Box Loss	Classification Loss	Distribution Loss	$\lambda_{box}$	$\lambda_{cls}$	$\lambda_{dfl}$
YOLOv8m–YOLOv12m	Ultralytics/PyTorch	CIoU	BCE	DFL	7.5	0.5	1.5

**Note:** YOLOv10 additionally retains its native dual-label assignment strategy, combining one-to-many and one-to-one supervision, without modifying the loss weighting coefficients reported in the table.

**Table 7:** Nodule-specific detection performance on the internal LUNG-PET-CT-DX validation set. Precision, Recall, F1-score, mAP@50, mAP@50–95, and inference time are reported for YOLOv8–YOLOv12 under the proposed anatomical-contextual framework. The table focuses exclusively on the Nodule class as the primary pathological target.

Model	Class	Precision (P)	Recall (R)	F1-Score	mAP@50	mAP@50–95	Inference Time (ms)
YOLOv8m	Nodule	0.976	0.919	0.947	0.982	0.890	11.08
YOLOv9c	Nodule	0.959	0.957	0.958	0.974	0.880	16.13
YOLOv10m	Nodule	0.979	0.935	0.956	0.987	0.835	9.25
YOLOv11m	Nodule	0.953	0.971	0.962	0.976	0.876	11.38
YOLOv12m	Nodule	0.942	0.968	0.955	0.977	0.832	12.58

**Table 8:** Ablation study evaluating the effect of anatomical context on pulmonary nodule detection using the internal LUNG-PET-CT-DX validation set.

Model	Training Scheme	Precision (P)	Recall (R)	F1-Score	mAP@50	mAP@50–95
YOLOv8m	Nodule-only	0.892	0.867	0.879	0.908	0.676
	**Nodule +** **Anatomical Context**	**0.976**	**0.919**	**0.947**	**0.982**	**0.890**
	Δ	+0.084	+0.052	+0.068	+0.074	+0.214
YOLOv9c	Nodule-only	0.871	0.823	0.846	0.903	0.661
	**Nodule +** **Anatomical Context**	**0.959**	**0.957**	**0.958**	**0.974**	**0.880**
	Δ	+0.088	+0.134	+0.112	+0.071	+0.219
YOLOv10m	Nodule-only	0.865	0.833	0.849	0.887	0.671
	**Nodule +** **Anatomical Context**	**0.979**	**0.935**	**0.956**	**0.987**	**0.835**
	Δ	+0.114	+0.102	+0.107	+0.100	+0.164
YOLOv11m	Nodule-only	0.890	0.806	0.846	0.894	0.665
	**Nodule +** **Anatomical Context**	**0.953**	**0.971**	**0.962**	**0.976**	**0.876**
	Δ	+0.063	+0.165	+0.116	+0.082	+0.211
YOLOv12m	Nodule-only	0.901	0.811	0.854	0.910	0.691
	**Nodule +** **Anatomical Context**	**0.942**	**0.968**	**0.955**	**0.977**	**0.832**
	Δ	+0.041	+0.157	+0.101	+0.067	+0.141

Note: Bold values indicate the results obtained using the Nodule + Anatomical Context training scheme.

**Table 9:** Nodule-specific performance during external evaluation on LIDC-IDRI using the Nodule + Anatomical Context training scheme. Results correspond to the Nodule class for YOLOv8–YOLOv12 models evaluated without retraining or fine-tuning.

Model	Training Scheme	Class	Precision (P)	Recall (R)	F1-Score	mAP@50	mAP@50–95	Inference Time (ms)
YOLOv8m	Nodule + Anatomical Context	Nodule	0.915	0.930	0.922	0.942	0.845	11.08
YOLOv9c			0.884	0.900	0.892	0.934	0.838	16.13
YOLOv10m			0.924	0.920	0.922	0.956	0.815	9.25
YOLOv11m			0.874	0.890	0.882	0.920	0.810	11.38
YOLOv12m			0.877	0.890	0.883	0.937	0.792	12.58

## Data Availability

The datasets used in this study are publicly available. LUNG-PET-CT-DX and LIDC-IDRI can be accessed through The Cancer Imaging Archive (TCIA). The processed data, annotations, and experimental files generated during this study are available from the corresponding authors upon reasonable request.
